# Genomic prediction of complex human traits: relatedness, trait architecture and predictive meta-models

**DOI:** 10.1093/hmg/ddv145

**Published:** 2015-04-26

**Authors:** Athina Spiliopoulou, Reka Nagy, Mairead L. Bermingham, Jennifer E. Huffman, Caroline Hayward, Veronique Vitart, Igor Rudan, Harry Campbell, Alan F. Wright, James F. Wilson, Ricardo Pong-Wong, Felix Agakov, Pau Navarro, Chris S. Haley

**Affiliations:** 1 MRC Human Genetics Unit, Institute of Genetics and Molecular Medicine, University of Edinburgh, Edinburgh EH4 2XU, UK,; 2 Pharmatics Limited, Edinburgh EH16 4UX, UK,; 3 Centre for Population Health Sciences, University of Edinburgh, Edinburgh EH8 9AG, UK and; 4 The Roslin Institute and Royal (Dick) School of Veterinary Studies, University of Edinburgh, Easter Bush Midlothian EH25 9RG, UK

## Abstract

We explore the prediction of individuals' phenotypes for complex traits using genomic data. We compare several widely used prediction models, including Ridge Regression, LASSO and Elastic Nets estimated from cohort data, and polygenic risk scores constructed using published summary statistics from genome-wide association meta-analyses (GWAMA). We evaluate the interplay between relatedness, trait architecture and optimal marker density, by predicting height, body mass index (BMI) and high-density lipoprotein level (HDL) in two data cohorts, originating from Croatia and Scotland. We empirically demonstrate that dense models are better when all genetic effects are small (height and BMI) and target individuals are related to the training samples, while sparse models predict better in unrelated individuals and when some effects have moderate size (HDL). For HDL sparse models achieved good across-cohort prediction, performing similarly to the GWAMA risk score and to models trained within the same cohort, which indicates that, for predicting traits with moderately sized effects, large sample sizes and familial structure become less important, though still potentially useful. Finally, we propose a novel ensemble of whole-genome predictors with GWAMA risk scores and demonstrate that the resulting meta-model achieves higher prediction accuracy than either model on its own. We conclude that although current genomic predictors are not accurate enough for diagnostic purposes, performance can be improved without requiring access to large-scale individual-level data. Our methodologically simple meta-model is a means of performing predictive meta-analysis for optimizing genomic predictions and can be easily extended to incorporate multiple population-level summary statistics or other domain knowledge.

## Introduction

Since the completion of the sequence of the human genome, accurate prediction of complex phenotypic traits from genotypes has become a central goal of biomedical research. Since genetic information remains largely unchanged through life, it is often argued that by applying our knowledge about the human genome to medical practice at the level of the individual patient, we can improve disease management in terms of prevention, diagnosis and treatment, thus optimizing the health care each individual receives (1,2) from as early as birth. For example, we can apply genetic testing to identify and monitor individuals with high genetic risk of disease, we can discriminate different subtypes of complex diseases, and we can do better targeting of treatments through pharmacogenomics. Promising findings from Genome-Wide Association Studies (GWAS) that are currently being translated into clinical use are discussed in a recent review by Manolio (3). These include increased prediction accuracy of Type 1 diabetes mellitus when comparing a model using all GWAS-identified genomic markers to a model using only markers in the Major Histocompatibility Complex (4), identification of C-reactive protein as a potential biomarker for the HNF1A-MODY monogenic form of diabetes (5), and better understanding and management of simvastatin-induced myopathy risk (6).

However, for most complex traits the adoption of gene-based personalized approaches to medical care remains limited. One reason is that clinical adoption of scientific discoveries can be slow in general. In the case of GWAS findings, clinical translation can be further prolonged by (i) the fact that the underlying biological mechanisms are often not well understood and (ii) the wide range of different expertise needed to initiate and implement genomic interventions (7,8). A second reason for the limited application of GWAS findings to medical care is the low predictive accuracy achieved by current genomic predictors of most complex human traits, including highly heritable traits such as height (9–11).

In human genetics, the task of genomic prediction has been most commonly addressed by considering results from GWAS. To make predictions, single-nucleotide polymorphisms (SNPs) that meet a predefined statistical significance threshold of association with the trait are considered and a *genetic score* is constructed by adding the estimated effects of the alleles each individual carries for these SNPs (12). In order to reduce the number of false-positive associations, a stringent significance threshold is typically applied, which means that only a dozen to a few hundred SNPs are considered in the genetic score. Furthermore, since the effect of each SNP is estimated separately, care must be taken to exclude SNPs that ‘tag’ the same causal locus (13). This can be performed by only considering the SNP with the lowest *P*-value within each region, or by pruning SNPs according to a predefined threshold on linkage disequilibrium (LD). Such heuristic approaches can be computationally expensive, and more importantly, are likely to include markers that are redundant, or exclude markers that carry complementary information to the already included ones, or both. GWAS-based genetic (or polygenic) risk scores have been used for prediction in a number of common diseases (12–15), and although some predictive ability is demonstrated, the accuracies achieved to date fall short of clinical utility.

A different approach to GWAS-based polygenic score constructions is to use statistical methods that estimate the effects for all SNPs jointly. Such approaches have been termed whole-genome prediction methods (16), and were first used as models for SNP data in genomic selection in animal breeding (17). An advantage of whole-genome prediction methods is that by construction they take into account LD between SNPs. Perhaps more importantly, the potential value of whole-genome predictors lies in their ability to *learn* what is important for the prediction task directly from the data, without the need to pre-select the input SNPs based on (typically univariate) measures of association with the trait. This property has become very important in light of the influential paper of Yang *et al*. (18), who demonstrated that by considering all the SNPs simultaneously, they could explain a much greater proportion of the trait heritability, than by considering only the few SNPs that pass the stringent association test.

There is a broad range of methods that can be used to implement whole-genome prediction (19). These often differ in terms of their prior assumptions regarding the distribution of SNP effects, which induce different estimation of model parameters. Here we are interested in comparing methods with respect to the number of genetic markers that are included in the resulting predictor, which we refer to as the *sparsity level*, with sparse models considering tens to a few hundred markers and dense models considering hundreds of thousands of markers. In the context of genomic prediction of complex traits, it is not yet clear when dense or sparse models are optimal. For genomic selection in animal breeding, de los Campos *et al*. (19) consider more than 50 studies that compare different estimation methods and conclude that, in empirical analyses of real data, there are only small differences between methods, with estimation methods leading to sparse models having a slight advantage. However, this relative performance is mainly based on livestock populations, which typically exhibit high LD and are genotyped using relatively sparse platforms (in the order of 50 000 SNPs), and hence may change when we examine genomic prediction of complex traits in humans, where LD is not as high and the genotyping platforms are denser. In plant breeding, Wimmer *et al*. (20) recommend that dense whole-genome models should be preferred over sparse ones, since most traits of agronomic performance are assumed to have many small genetic effects, and medium trait heritabilities, while the genomic data exhibit a large extent of LD.

In human genetics, two recent studies (21,22) have compared dense with sparse models using different whole-genome estimation methods and polygenic score constructions. Abraham *et al*. (21) find that model performance depends on the genetic architecture of the trait, with sparse whole-genome estimation methods being better than dense estimation methods and polygenic scores when modelling traits with large genetic effects concentrated in a few genomic regions, while all methods perform similarly when predicting traits with no known large genetic effects. Warren *et al*. (22) compare dense and sparse shrinkage methods with polygenic scores in predicting two lipid traits with similar genetic architectures. They find that sparse estimation methods achieve lower mean squared error than dense ones, when all the SNPs are given as input to the model, but different estimation methods have similar results when they select the optimal number of markers using a validation set.

In this work, we compare dense and sparse genomic prediction models with respect to trait genetic architecture and population structure. Specifically, we consider different scenarios regarding relatedness between training samples and the target population and evaluate which models have good generalization performance within a single cohort (related individuals) and across different cohorts (unrelated individuals), and what are the characteristics of the trait that make such generalization possible. In human genetics, the accuracy of genomic prediction is often assessed using nominally unrelated individuals. However, familial structure is an important source of predictive signal, with relatedness between training and target samples being one of the most important factors affecting predictive performance both in animal breeding (19) and in human complex traits (23–25). As more individuals are genotyped and summary statistics from different cohorts are aggregated to increase statistical power, familial relationships for an increasing number of individuals will be available and hence there is a need to learn how to best utilize this signal. To our knowledge, this is the first study that examines the interplay between relatedness, trait genetic architecture and optimal model sparsity in the prediction of complex traits in humans.

Furthermore, we evaluate the predictive value of genome-wide association meta-analyses (GWAMA) by comparing the performance of models estimated from our data cohorts to that of a polygenic risk score constructed using only GWAMA ‘hits' and their GWAMA estimated effects, which are based on more than 100 000 individuals. Our final contribution is the construction and evaluation of a simple meta-model that combines dense whole-genome predictors with GWAMA-based polygenic risk scores. Current limitations in genomic prediction of complex traits include incomplete genomic information (26,27), which means that some causal loci are poorly tagged by the genotyped SNPs, and lack of statistical power to detect true associations (26,28,29), which means that the predictive signal is difficult to separate from the data noise. Our meta-model partly addresses the first limitation by exploiting the familial structure captured by dense whole-genome predictors, and increases the statistical power through the GWAMA risk score, which is based on summary statistics from the largest sample size currently available.

## Results

In the following sections, we evaluate and compare a number of different genomic predictors in within and across-cohort prediction and explore how we can increase prediction accuracy by combining models, or optimizing our experimental design. We consider a GWAMA-based polygenic score and three penalized regression methods, Ridge Regression (RR) (30), the Least Absolute Shrinkage and Selection Operator (LASSO) (31) and the Elastic Net (EN) (32). RR results in dense models, while LASSO and EN can result in sparse models by assigning a zero coefficient to some markers. The penalized regression methods are applied using all available genotyped markers (267 912 SNPs) or subsets of the genotyped markers selected based on GWAS pre-filtering (using our population data). The GWAMA-based scores are sparse by construction—we only include the SNP ‘hits’ from the published GWAMA study for each trait (33–35). These SNP ‘hits’ are significantly associated with the trait at the genome-wide significance threshold and LD is taken into account, typically by reporting only the most significant association within each genomic region. For SNP ‘hits’ that are not directly genotyped in our data, we use imputed dosages when constructing the GWAMA-score. To quantify prediction accuracy, we use the Pearson's correlation coefficient between the true and the predicted phenotypes. We use three types of data sets in our experiments. The *training* data are used for parameter estimation, the *validation* data are used for model selection (e.g. to select the optimal penalty strength in the penalized regression methods) and the *testing* data are used to estimate prediction accuracy. Throughout this article, we always report prediction accuracy on the *testing* data. More details are given in Materials and Methods.

**Figure 1.**
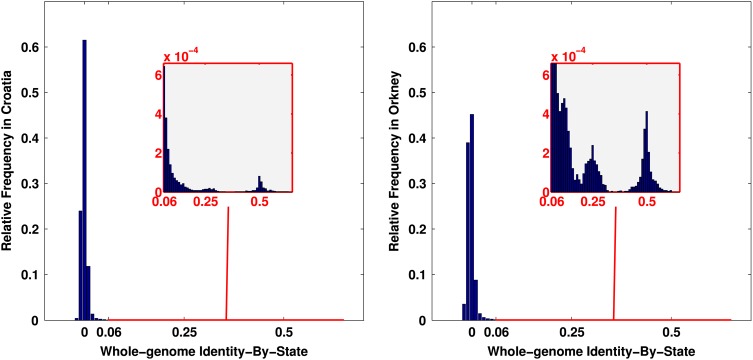
Whole-genome Identity-By-State coefficient of relationship between individuals in Croatia (left) and in Orkney (right). Each panel shows a histogram of the genetic similarity between every pair of individuals from each data set. The sub-panels display in more detail the right tail of the histogram by zooming-in on the y-axis. The similarity between individuals *i* and *k* is computed by $s_{ik}=\frac{1}{P}\sum_{\;j=1}^{P}\frac{\left(x_{ij}-2\;p_{j})(x_{kj}-2\;p_{j}\right)}{2\;p_{j}(1-\;p_{j})},$ where *p_j_* is the minor allele frequency (MAF) of SNP *j*, $x_{ij}\in \{0,1,2\}$ is the genotype of individual *i* at SNP *j*, and *P* = 267 912 (260 562 in Orkney) is the number of the genotyped SNPs. Both cohorts contain related individuals, demonstrated in the sub-panels by the density mass around 0.5 (expected IBS between parent-offspring and full siblings), 0.25 (expected IBS between half siblings and uncle/aunt with nephew/niece) and 0.125 (expected IBS between first cousins). The proportion of related individuals is higher in Orkney compared with Croatia, demonstrated by the higher density in IBS values corresponding to familial relationships.

We examine two data sets, namely Croatia and Orkney. These differ in terms of familial and ancestry population structure. The Croatian data set comprises individuals from three studies performed at three locations—two Dalmatian islands and one mainland coastal city. The Orkney data set comprises individuals from a single study, recruited with an emphasis on co-recruiting family members. More details about the data cohorts are given in Materials and Methods. The genomic relatedness in Croatia and Orkney, estimated from the available 267 912 genotyped SNPs, is presented in Figure 1. The proportion of familial to non-familial relationships is higher in the Orkney cohort than it is in the Croatian cohort.

We examine three complex traits, namely height, body mass index (BMI) and high-density lipoprotein (HDL) cholesterol. The genetic contribution to complex phenotypic traits is typically analysed in terms of trait *heritability*, i.e. the amount of phenotypic variance that can be attributed to genetic effects, and in terms of *genetic architecture*, which describes the number, effect sizes, allele frequencies and modes of action of the genetic variants affecting a trait, and is difficult to infer in complex traits and thus largely unknown. Current heritability benchmarks suggest high, low and moderate contributions of genetic effects in height, BMI and HDL, respectively (36). Additionally, empirical evidence emerging from GWAS and GWAMA studies—which test trait association with common SNPs—suggests that these three traits differ in terms of the distribution of effect sizes for the ‘interrogated’ genetic variants. More specifically, although the evidence is suggestive of a polygenic architecture for all three traits, in height and BMI all the GWAMA-estimated SNP effects have small magnitude, whereas in HDL there is a small number of SNPs with moderate effect sizes in addition to a large number of SNPs with small estimated effect sizes (33–35).

Heritability estimates for the three traits are given in Table 1. The first two columns show heritability estimates using all the samples in each of our two cohorts (both related and unrelated individuals) and are computed using a linear random-effects model and all available genotyped SNPs. The last two columns show heritability estimates reported in the literature. Both reported estimates use common SNPs across the whole genome; the first one is computed using only related individuals, and thus can be interpreted as a proxy for heritability estimated in family studies, while the second one is computed using only unrelated individuals. Due to the existence of familial relationships in our data cohorts, our genomic heritability estimates are close to the reported ‘family-based’ heritability of each trait, which is higher than the heritability captured by common SNPs in cohorts of unrelated individuals.

**Table 1.** Heritability estimates in our cohort data and reported in the literature

Trait	$\hat{h_{SNP,Croatia}^{2}}$	$\hat{h_{SNP,Orkney}^{2}}$	$\hat{h_{related,pub}^{2}}$	$\hat{h_{unrelated,pub}^{2}}$
Height	0.81	0.90	0.88 (0.09) (36)	0.46 (0.05) (36)
BMI	0.29	0.56	0.34 (0.12) (36)	0.14 (0.05) (36)
HDL	0.57	0.61	0.48 (0.11) (36)	0.12 (0.05) (36)
$\hat{h_{SNP,Croatia}^{2}}$: Estimated using all 267 912 genotyped SNPs and a linear random-effects model (polygenic function in the GenABEL R package). Each trait is adjusted for the effects of sex, age and age^2^ and the residuals are then *z*-transformed. HDL is additionally log-transformed as a first step (i.e. before adjusting for the effects of sex, age and age^2^). This pre-processing is performed separately for each of the three Croatian sub-cohorts (Korcula, Vis and Split) and the transformed phenotypes are then considered jointly in the linear random-effects model.				
$\hat{h_{SNP,Orkney}^{2}}$: Estimated using all 260 562 genotyped SNPs and a linear random-effects model (polygenic function in the GenABEL R package). Each trait is adjusted for the effects of sex, age and age^2^ and the residuals are then *z*-transformed. HDL is additionally log-transformed as a first step (i.e. before adjusting for the effects of sex, age and age^2^).				
$\hat{h_{related,pub}^{2}}$: Estimated using common SNPs and 530 related individuals from the ARIC study. Individuals were selected to have at least one relative in the data, and relatives were defined as having genome-wide SNP similarity between 0.35 and 0.65, derived empirically [see (36) for details].				
$\hat{h_{unrelated,pub}^{2}}$: Estimated using common SNPs and 5647 unrelated individuals from the ARIC study, with genome-wide SNP relationships below 0.025 [see (36) for details].				

Histograms of GWAMA-estimated SNP effects for each of the three traits are presented in Supplementary Material, Figure S1. The fat right tail of the histogram for HDL illustrates the existence of some SNPs with moderate effect sizes.

### Within-cohort prediction

**Figure 2.**
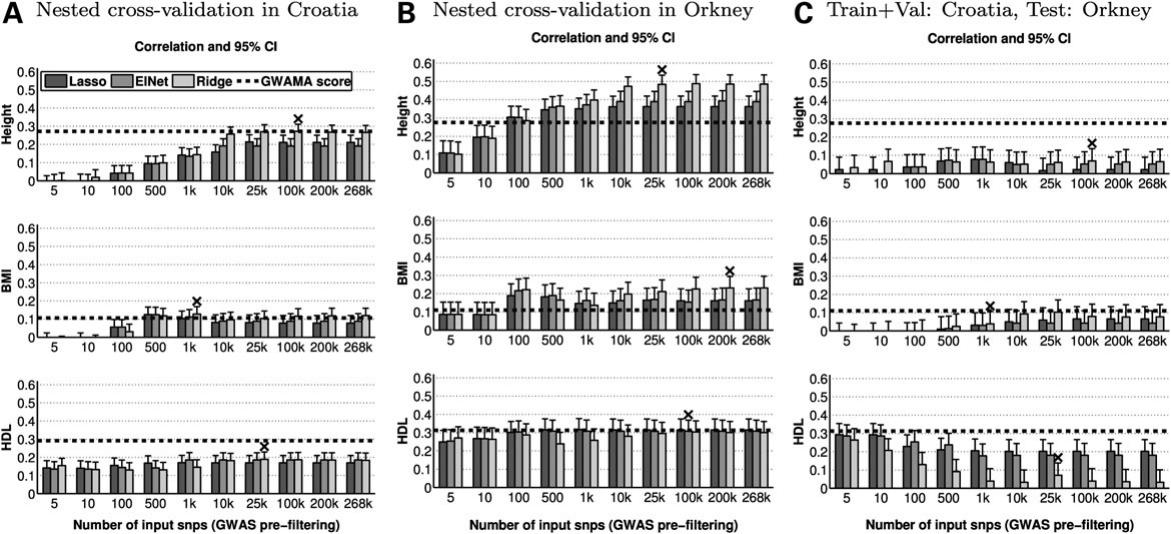
Accuracy of penalized regression models as we increase the number of input SNPs together with accuracy of the GWAMA-based polygenic score. (**A**) Within-cohort prediction in Croatia. (**B**) Within-cohort prediction in Orkney. (**C**) Across-cohort prediction (training & validation: Croatia, testing: Orkney). y-axis: accuracy is measured using the Pearson's correlation coefficient between the predicted and true phenotype of the testing samples. The error bar corresponds to the 95% Confidence Interval (upper CI). x-axis: an increasing number of SNPs (genetic markers) are given as input to the regression models. The SNP selection is performed by GWAS pre-filtering using the training data in each case. The number of input SNPs for the GWAMA-based risk score is constant (height: 180 SNPs, BMI: 32 SNPs, HDL: 70 SNPs). *Black ‘x’ symbol*: depicts the optimal penalized regression model—in terms of the type of shrinkage penalty and the number of input markers—based on prediction accuracy on the validation set. This corresponds to the model that we would select as our best predictor in a real-world application.

Figure 2A and B show accuracy (correlation coefficient and 95% confidence interval) in within-cohort prediction from three penalized regression models—LASSO, EN and RR—as we increase the number of SNPs that are given as input to the model. The input SNPs are selected using the GWAS-based pre-filtering procedure (see Materials and Methods). The plots in (A) show prediction accuracy when the training, validation and testing steps are performed using samples from the Croatian populations (nested cross-validation design), while the plots in (B) show prediction accuracy when the training, validation and testing steps are performed using individuals from Orkney (nested cross-validation design). Rows correspond to different traits.

First, we want to assess if prediction of complex traits is possible using data cohorts with a few thousand individuals. The penalized regression model (type of model and number of input SNPs) that achieves the highest accuracy in the validation step is depicted by a black ‘x’ symbol in Figure 2. This optimal model from the validation step also achieves the highest accuracy on the testing data for all traits and both populations. Furthermore, performance of RR trained using all 267 912 available SNPs is always close or equal to that of the best within-cohort predictor. Therefore, performance in this set of experiments can be maximized by either selecting the optimal penalty type and number of input markers through a validation step, or by choosing a priori a dense, whole-genome predictor. The absolute value of the correlation coefficient between true and predicted phenotypes for the optimal penalized regression model is related to the heritability of the trait, which is consistent with the literature (25,26). In both populations, the accuracy achieved for height is higher than the accuracy achieved for HDL, which is in turn higher than the accuracy for BMI, and height has the highest estimated heritability followed by HDL and then BMI. Furthermore, the level of accuracy achievable by penalized regression methods depends on the population structure, and more specifically on the extent of familial structure in the data. As illustrated in Figure 1, the proportion of familial relationships is higher in the Orkney data set than it is in Croatia, and in Figure 2, the accuracy of the optimal penalized regression model is higher in Orkney than it is in Croatia for all three traits. Additionally, the relatedness between training and testing samples resulting from the nested cross-validation split is as follows: in Orkney 88% of the testing samples have at least one nominal relative in the training data—genome-wide Identity-By-State (IBS) similarity ≥0.1—as opposed to 52% in Croatia.

Comparing the different penalized regression models, we can see that in within-cohort prediction, dense models (RR or many input and retained SNPs) tend to outperform sparse models (LASSO and EN or few input SNPs). In all cases, the performance of penalized regression models using the top 5, 10 and in most cases 100 SNPs (ranked by GWAS *P*-value in the training data) is lower than that of denser models using thousands of SNPs. This difference is statistically significant in height and BMI, based on a Fisher *z*-transformation of the correlation coefficient (see Materials and Methods). On the other hand, the performance of sparse penalized regression models is comparable and occasionally better to that of dense models in HDL. Additionally, in all traits and both populations there is a plateau in performance with respect to the number of input SNPs; after we have included the top 10 000 to 25 000 SNPs, further increasing the number of input SNPs does not lead to an increase in prediction accuracy. Interestingly, the optimal penalized regression model—selected based on performance on the validation data—is always RR with at least 1000 SNPs and in 5 out of 6 cases more than 25 000 SNPs, which correspond to dense whole-genome predictors after we have performed the initial GWAS-based filtering of markers.

Next, we compare the accuracy of within-cohort prediction to the accuracy achieved by the GWAMA-based polygenic score. The optimal penalized regression model performs equally well or better than the GWAMA-based polygenic score in many cases, and we empirically observe that this difference in performance depends on the trait architecture and the level of relatedness in the study cohort. The optimal penalized regression model significantly outperforms the GWAMA-based polygenic score when predicting height and BMI in Orkney, and achieves similar accuracy to the polygenic score when predicting height and BMI in Croatia, suggesting that in traits with many small effects, the familial structure may be better at representing the predictive signal in the data, compared with the GWAMA hits. On the other hand, the GWAMA score significantly outperforms the optimal penalized regression model when predicting HDL in Croatia, and the two methods have similar accuracy in Orkney. This suggests that when the trait has at least some SNPs with moderate genetic effects, then the GWAMA score could be advantageous over prediction models estimated from single-cohort data, as common SNPs with large or moderately sized genetic effects are easier to identify and estimate accurately in a GWAMA.

An interesting question regarding prediction accuracy, especially in the case of height and BMI, is what happens if we use a genetic predictor based solely on pedigree information from our two cohorts. We construct a SNP-based proxy for pedigree by applying a threshold to the genome-wide IBS similarity matrix and setting relationships smaller than the threshold to zero, thus only retaining relationships above a certain coefficient of relatedness. Results using this SNP-based pedigree are shown in Supplementary Material, Table S1. The SNP-based pedigree leads to decreased performance compared with the optimal penalized regression model, and performance becomes worse as we apply higher thresholds on the IBS similarity matrix, i.e. as we restrict the SNP-based pedigree to higher degrees of familial relationships.

**Figure 3.**
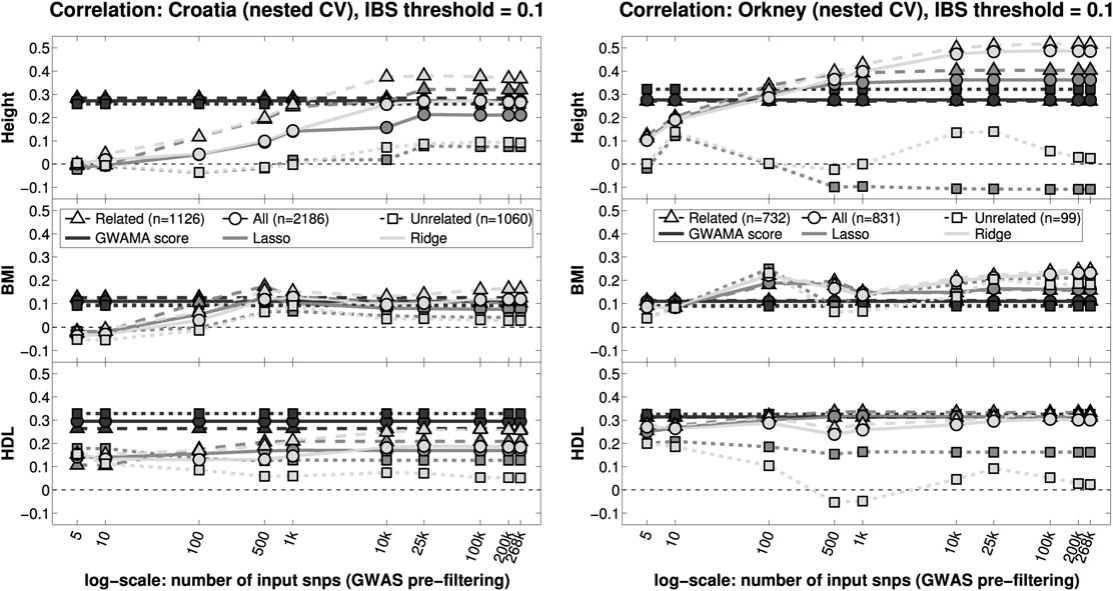
Accuracy of LASSO and RR in within-cohort prediction computed using ‘related’, ‘unrelated’ and ‘all’ testing individuals (IBS threshold = 0.1) together with accuracy of the GWAMA-based polygenic score for the same individuals. The ‘related’ group (*triangles*) contains testing individuals with at least one nominal relative in the training data (IBS ≥ 0.1). The ‘unrelated’ group (*squares*) contains testing samples that are nominally unrelated to all the training samples. The ‘all’ group (*circles*) contains all the testing samples. *y-axis*: accuracy is measured using the Pearson's correlation coefficient between the predicted and true phenotype of the corresponding testing samples. *x-axis*: an increasing number of SNPs are given as input to the regression models (plotted in log-scale). The SNP selection is performed by GWAS pre-filtering using the training data in each case. *Left*: training, validation and testing are performed using samples from the Croatia dataset (nested cross-validation design). *Right*: training, validation and testing are performed using samples from the Orkney dataset (nested cross-validation design). Performance in the ‘related’ group is always better than that in the ‘unrelated’ group for LASSO and RR and this difference increases as we consider denser models. Performance of the GWAMA score is very similar in the ‘related’ and the ‘unrelated’ groups. The accuracies computed in ‘related’ and ‘unrelated’ groups based on a smaller IBS threshold (IBS threshold = 1/16) are given in Supplementary Material, Figure S8.

To further empirically explore the effect of familial relationships between training and testing samples on the performance of penalized estimation methods, we split the testing data in two groups, individuals with at least one nominal relative (IBS ≥ 0.1) in the training data (referred to as ‘related’) and individuals with no relatives in the training data (referred to as ‘unrelated’) and compute prediction accuracy in each group. The results from this analysis for LASSO and RR are presented in Figure 3, together with the predictive correlation of the GWAMA score to facilitate comparison. Performance in the ‘related’ group is always better than that in the ‘unrelated’ group for LASSO and RR and this difference increases as we consider denser models, i.e. RR compared with LASSO, and many input SNPs compared with fewer input SNPs. On the other hand, the performance of the GWAMA score is very similar in the ‘related’ and the ‘unrelated’ groups.

Figure 3 suggests that the predictive signal in the dense regression models is different to the predictive signal in the GWAMA-based polygenic score method, with the former capturing mostly familial structure and the latter capturing genetic effects that generalize across many European populations by construction. Our next analysis aims to assess whether we can achieve better prediction accuracy by combining these two sources of predictive signal. To this end, we construct a meta-model by taking a linear combination of the two predictors, namely the predicted value from the optimal penalized regression model and the GWAMA-based polygenic score (see Materials and Methods).

**Figure 4.**
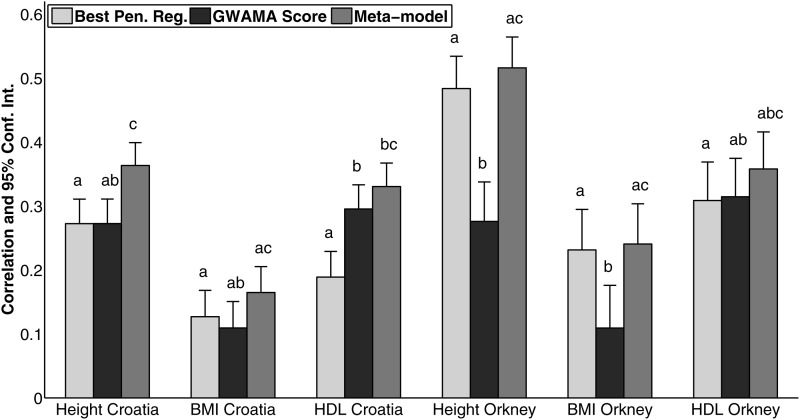
Accuracy of the optimal penalized regression model, the GWAMA-based polygenic score and the meta-model combining the two. y-axis: accuracy is measured using the Pearson's correlation coefficient between the predicted and true phenotype of the testing samples (nested cross-validation design for best penalized regression model, doubly nested cross-validation design for the meta-model). The error bar corresponds to the 95% Confidence Interval (upper CI). abc: denotes statistical significance from a one-tailed paired *z*-test comparing model performance. Superscript *a* denotes that a model is not statistically different to the first bar of the group, while superscript *b* denotes that a model is not statistically different to the second bar of the group. The type of shrinkage and number of input SNPs for the optimal penalized regression model in each case are given in Table 3.

The prediction accuracy of the meta-model is shown in Figure 4, together with the prediction accuracy of the GWAMA score and of the optimal penalized regression model. The prediction accuracy achieved by the meta-model is always higher than that achieved by either the polygenic score or penalized regression. Furthermore, the performance achieved by the meta-model is often statistically significantly better compared with the performance of its components, where statistical significance is tested using a Fisher *z*-transformation of the correlation coefficient.

Finally, we wanted to assess if performance in within-cohort prediction improves if we increase the sample size of our training data by including individuals from a different population cohort. Results from within-Orkney prediction where we include individuals from Croatia in training are shown in Supplementary Material, Figure S2C. There are no statistically significant changes between this experiment and the Orkney within-cohort experiment presented in Figure 2B. However, we can note that for all three traits the highest prediction accuracy is achieved when we use the combined training data set, which suggests that it could be beneficial to train our predictive models using all available cohorts, even if some of them are less closely related to the target population.

### Across-cohort prediction

In this section, we examine which models can achieve good generalization performance in across-cohort prediction, i.e. when the target individuals come from a different cohort (population) to the training samples. Figure 2C shows the prediction accuracy (correlation coefficient and 95% confidence interval) of three penalized regression models—LASSO, EN and RR—when the training and testing data come from south-east and north-west Europe, respectively. Again, we evaluate performance in models with an increasing number of input SNPs, selected by performing GWAS pre-filtering on the training data. In all the subfigures, the models are trained using all the individuals from Croatia and prediction accuracy is measured by considering the predicted and true phenotypes in all individuals from Orkney. The strength of the shrinkage penalty, i.e. the meta-parameter that controls how much shrinkage is applied on the model parameters, is selected based on samples from Croatia. Specifically, we use the penalty strength that maximizes the predictive correlation in within-cohort prediction in Croatia. An alternative way of selecting the penalty strength is presented in Supplementary Material, Figure S2B, where we split the Orkney samples in 10-folds and iteratively use 9-folds to select the optimal penalty strength (validation) and make predictions on the held-out fold (testing). See Materials and Methods for more details.

The penalized regression models trained using individuals from Croatia are poor predictors for height and BMI in Orkney. Irrespective of whether we use a dense or a sparse model, prediction accuracy for height and BMI is low in across-cohort prediction. Indeed, we cannot reject the null hypothesis of no correlation between the true and the predicted phenotypes for any of the penalized regression models with 99% confidence. For some models, we can reject the null hypothesis with 95% confidence, but overall, the penalized regression models for height and BMI trained using individuals from Croatia do not have good generalization performance in the Orkney data set. In contrast, in across-cohort prediction of HDL (Fig. 2C, bottom row) the accuracy of sparse models is statistically different to zero, and significantly higher than that of denser models (RR with many input SNPs or LASSO and ENs with many SNPs retained in the solution). Additionally, the across-cohort prediction accuracy of sparse models is not statistically different to that achieved by the GWAMA-based polygenic score method in HDL, despite the fact that the former is trained using a 100 times fewer samples than the latter.

Furthermore, for across-cohort prediction of HDL, the performance of RR becomes substantially worse as we increase the number of input SNPs, while the performance of LASSO and EN has a smaller decline to RR as we increase the number of input SNPs from 5 to 500, and has no further decline as we add more input SNPs. Interestingly, when the validation samples (used to select the optimal penalty strength) come from Orkney (Supplementary Material, Fig. S2B), prediction accuracy of the sparse estimation methods—namely LASSO and EN—remains the same irrespective of the number of input SNPs. The LASSO and EN models that achieve the optimal predictive correlation are very sparse, with the number of retained SNPs being 3 and 4 for the LASSO and EN, respectively. The retained SNPs in LASSO are *rs3764261*—most significant hit in the HDL GWAMA, and also used in the polygenic score (GWAMA *P*-value = 1 × 10^−769^), *rs7499892*—significant in the HDL GWAMA, but not reported in the paper as it is in the same region as *rs3764261* (GWAMA *P*-value = 1 × 10^−541^), and *rs1532085*—4th most significant hit in the HDL GWAMA, and also used in the polygenic score (GWAMA *P*-value = 1 × 10^−188^).

## Discussion

### Dense whole-genome predictors and relatedness

Our main goal is to compare different dense and sparse genomic prediction models with respect to the genetic architecture of the trait and the population structure of the study sample, primarily in terms of relatedness. In our first set of experiments, we showed that in within-cohort prediction it is possible to exploit familial structure and get good prediction accuracy using dense whole-genome predictors. The dense whole-genome predictors act as proxies of pedigree information and can often achieve higher prediction accuracy than the GWAMA-based polygenic score, despite using two orders of magnitude fewer samples (<3000 samples versus >100 000 samples, respectively) and lower coverage of the genome (<268 000 genotyped SNPs versus >2 000 000 SNPs tested for association in GWAMAs, respectively). It is interesting to note that <30% of the GWAMA reported significant SNPs are genotyped in our data sets (22 out of 180 in height, 7 out of 32 in BMI and 20 out of 70 in HDL). This means that only a subset of the GWAMA ‘hits’ is directly given as input to the penalized regression models, since we only use genotyped SNPs as inputs in this case. On the other hand, the majority of GWAMA ‘hits’ are used as inputs in the GWAMA-based scores, since in this case we use imputed dosages for GWAMA ‘hits’ that are not directly genotyped, and we have imputed dosages with relatively good imputation quality for all but 3 of the GWAMA ‘hits’ (Supplementary Material, Table S2).

The within-cohort prediction accuracy of dense whole-genome predictors is higher when we have closer familial relationships between training and testing samples, with accuracy in Orkney being higher than accuracy in Croatia for all three traits (Fig. 2A and B). This is in accordance with Legarra *et al*. (37), who first demonstrated the predictive value of familial structure in genomic prediction by considering full-sib families of mice and showing that accuracy of whole-genome predictors was higher when each family was split in half between training and testing data compared with accuracy when each family was put as a whole either in training or in testing data.

The good performance of dense models in within-cohort prediction is most likely due to their ability to capture familial structure in the data that is predictive of the trait. The effect of relatedness on prediction accuracy is illustrated in Figure 3 where the predictive correlation of denser models (>100 input SNPs) is higher when estimated using testing individuals that have at least one relative in the training data (whole-genome IBS ≥ 0.1) than when estimated using individuals that are nominally unrelated to the training samples (whole-genome IBS < 0.1). This difference in accuracy estimated from the ‘related’ and the ‘unrelated’ groups is higher in Orkney than it is in Croatia and it is higher in denser models (RR, many input SNPs) than it is in sparser ones (LASSO, few input SNPs). When we examine the cross-validation folds, Orkney has both a higher proportion of testing samples with at least one relative in the training data and a higher proportion of testing samples that are related to multiple training samples compared with Croatia (Supplementary Material, Fig. S3). The higher prediction accuracy that we observe in Orkney could therefore be due to the increased relatedness between training and testing individuals, which would be consistent with the analytical expressions derived in (25) to quantify the impact of relatedness between training and testing individuals on the prediction accuracy of G-BLUP (equivalent to RR). The familial structure tends to be more informative in traits with many small effects and high heritability, as in these cases the similarity at the genotyped SNPs tends to be more informative of the (potentially unobserved) causal loci in related individuals compared with unrelated individuals.

Nevertheless, our results using SNP-based pedigree predictors (Supplementary Material, Table S1) suggest that the data from non-relatives can carry additional predictive information, which is utilized by the dense whole-genome regression models. Specifically, the optimal penalized regression model performs better than the SNP-based pedigree predictors for all three traits and both populations, and restricting the SNP-based pedigree to increasingly more closely related individuals leads to reduced prediction accuracy in most cases. In this work, we have not examined prediction based on recorded pedigree, but we note that SNP-based whole-genome predictors have previously been shown to outperform genealogy models in humans (24,38). This has mainly been attributed to two factors. First, recorded pedigrees for human cohorts are typically sparse, and therefore leveraging predictive signal from the unrelated individuals is helpful. This is also supported by our comparison of dense whole-genome models to SNP-based pedigree predictors. Secondly, similarity based on SNP markers across the genome expresses the *realized* genetic relationship between individuals, while similarity based on a recorded pedigree expresses the *theoretical expectation* for the genetic relationship between individuals. The former can be more informative of the realized relationship at the causal loci in related individuals and thus more predictive of the trait.

Finally, we note that relatedness between individuals in the training data can also have an impact on prediction accuracy. For instance, if we have genetic effects that only occur within a family (e.g. due to family specific rare variants), then including multiple family members in the training data can lead to a model that represents such effects. These effects will then generalize to other members of the family, but will not generalize to individuals outside the family. Hence, the difference in accuracy estimated from the ‘related’ and the ‘unrelated’ groups in Figure 3 could also be due to a shift in the distribution of effect sizes between the training data and the testing data in the ‘unrelated’ group.

### Pre-filtering

Overall, if we rely on the signal from familial structure to drive prediction, then a dense, whole-genome linear predictor, such as RR or G-BLUP with all the available SNPs as input, is a good model choice, especially for traits that have many small genetic effects and high heritability, such as height. This observation is demonstrated in Figure 2A and B. For all traits and both populations, RR with all 267 912 input SNPs is either the best or statistically not different to the optimal penalized regression model, and in height and BMI it performs better than sparser counterparts. However, we can also notice that RR models with 50 000 SNPs—or fewer depending on the population and the trait—are statistically not different to RR models with all available SNPs in within-cohort prediction. Therefore, if it is beneficial for the final application, e.g. if we want to apply algorithms that scale with respect to the dimensions, or if we want to genotype target individuals at lower coverage, we could perform feature selection as a first step and discard SNPs that are uninformative of the phenotype, for instance SNPs with an association *P*-value > 0.8.

### Meta-model

A key contribution of our work is the development and evaluation of a methodologically simple meta-model, which improves prediction by combining two types of genomic signal, the GWAMA-based risk score and a dense whole-genome predictor estimated from our population data. When considering the genetic architecture of complex traits, we can contemplate a range of possible genetic effects in terms of effect size, allele frequency and mode of action. For instance, we can have alleles that are common across multiple, potentially admixed, populations and have large or moderately sized additive effects on a trait. These are the easiest to identify, estimate and replicate by GWAS. We can have alleles with very small effects on a trait, or alleles that are less common, though still present in multiple populations. Such genetic effects are more difficult to detect by GWAS, but can be identified by GWAMAs with an increasing number of samples. However, we can also have alleles that only appear in a single population or even a single family, e.g. due to recent mutations, or alleles that only affect a subset of the individuals that carry them, e.g. due to interaction with a specific environment or interaction with a certain genetic background. Our meta-model demonstrates that prediction accuracy can be improved by combining predictors that capture different parts of the genomic signal, i.e. different types of genetic effects.

By construction, the GWAMA risk score captures genetic effects that are large enough to meet the statistical significance criteria in a genome-wide scan and hence, in principle, generalize across European ancestry populations, even if their absolute effect sizes differ among populations. On the other hand, the dense penalized regression models, whereby dense refers to models with more than 1000 retained SNPs, capture population (relatedness) structure that is predictive of the trait. The predictive signal in this case is similar to that of a pedigree-based predictor, which encodes the expected similarity of each pair of individuals at every locus. In related individuals, due to sharing of long genetic segments, similarity at the observed genetic loci is more informative about similarity at the (unobserved) causal loci compared with non-relatives, and hence more informative of the phenotypic similarity [this is explored in more detail by (25)]. We also note that, similar to pedigree-based predictors, dense whole-genome predictors could be capturing predictive signal that is due to factors besides linear additive genetic effects, such as dominance effects and epistasis (39).

By combining the GWAMA-based polygenic score with a dense whole-genome predictor, the meta-model represents both the summary statistics from large GWAMAs and the population-specific statistics from our data cohorts and always performs better than either model on its own (Fig. 4). Alternative ways of combining these two sources of information exist. For instance, we could fit a whole-genome regression model that treats the GWAMA SNPs as fixed effects, and the remaining SNPs as random effects. Although other approaches to building on prior knowledge are definitely worth exploring, we note that many of the alternatives will depend on the choice of predictive models and resulting optimization algorithms. In contrast, our meta-model is generic and easily extendible to a broad range of predictive models trained in possibly different cohorts. A practical implication of this is that we can improve the accuracy of genetic predictors without the need to access large-scale individual-level data sets, and we can potentially combine multiple predictors based on different summary statistics, e.g. combine risk scores based on multiple GWAS studies if a GWAMA is not available.

### Across-cohort generalization

In contrast to within-cohort prediction, in across-cohort prediction sparse models have better generalization performance. Results from large GWAMAs are therefore key if we want to construct predictors that are widely applicable, especially for traits that have many, small genetic effects such as height and BMI. Nevertheless, our results for across-cohort prediction of HDL (Fig. 2C and Supplementary Material, Figure S2B) suggest that—subject to the trait genetic architecture—good generalization performance in across-cohort prediction is possible even with a small training set. In contrast to height and BMI, where all genetic associations with common SNPs have small effect size, in HDL we find a few common SNPs that have moderate effect sizes, in addition to a potentially large number of genetic variants with small effect sizes (Supplementary Material, Fig. S1). The moderately sized effects are easier to capture in the Croatian data and separate from other structure that does not generalize to Orkney (such as familial predictive signal), compared with small effects that could generalize to Orkney, but are ‘swamped’ by other structure in the data. Generally, the ability of a model to generalize to new populations and nominally unrelated individuals will depend on the trait architecture; for a finite, relatively small, number of training samples, the larger and fewer the genetic effects associated with a trait the easier it will be for a model to separate this predictive signal from other data structure. Additionally, generalization performance will depend on the suitability of the model with respect to the target population. As we try to predict increasingly more distantly related individuals—from ‘related to the training data’ to ‘nominally unrelated but from the same geographic location’ to ‘nominally unrelated and from a different geographic location’—we expect that fewer effects will generalize, and thus sparser models are likely to perform better. Denser models will tend to fit the training data more closely, but some of this captured signal is likely to be irrelevant in a distantly related population, thus introducing noise in the prediction, which can be interpreted as a form of over-fitting. For instance, in the case of HDL, the very sparse models (<5 SNPs in the solution) capture only the moderately sized genetic effects. The denser models include these genetic effects, but also capture predictive familial structure from the Croatian data set, which does not generalize to Orkney.

Our results in across-cohort prediction of HDL are consistent with (22) who found that sparse models (including from 20 to 100 SNPs) were optimal for genomic prediction of HDL in two cohorts comprising unrelated individuals. In our experiments, the optimal models for across-cohort prediction of HDL are sparser (including from 3 to 5 SNPs). This can be due to the difference in the available genotyped SNPs in the two studies, the increased power of the study in (22) to distinguish SNPs tagging causal loci with smaller effects [the cohorts in (22) are larger and comprise unrelated individuals, which reduces other types of predictive signal in the training data], or a difference in minor allele frequencies (MAFs) in the different cohorts. Indeed the MAFs of the three SNPs retained in the optimal LASSO model are higher in Orkney than in Croatia (Table 2) and thus we can expect that a larger proportion of phenotypic variation will be attributable to these three SNPs in Orkney. This can also explain why the 3-SNP LASSO model achieves higher prediction accuracy in Orkney than in Croatia (Figure 2A and C), even though it is trained using samples from Croatia. Overall, we expect that the optimal number of input SNPs may increase with larger sample sizes, as we have more power to detect and estimate genetic effects. However, we also expect that there is a global optimum for the number of input SNPs, which is trait-depend.

**Table 2.** MAFs of the three SNPs retained in the LASSO model with the highest accuracy in across-cohort prediction of HDL

Rsid	MAF in Orkney	MAF in Croatia
rs3764261	0.3909	0.3138
rs1532624	0.4922	0.4099
rs7499892	0.2292	0.1631

The three SNPs have a higher minor allele frequency (MAF) in Orkney compared with Croatia and thus we expect that more phenotypic variance will be explained by these three SNPs in Orkney.

**Table 3.** Type of shrinkage and number of input SNPs for the optimal penalized regression model in within-cohort prediction

	Height Croatia	BMI Croatia	HDL Croatia	Height Orkney	BMI Orkney	HDL Orkney
Type of shrinkage	Ridge	Ridge	Ridge	Ridge	Ridge	Elastic Net
Number of input SNPs	100 000	1000	25 000	25 000	200 000	100 000

Finally, our results in across-cohort prediction of HDL illustrate the importance of the validation data set. In the sparse models (LASSO and EN) if we select the penalty strength using a held-out set of individuals from the target population at the validation step, then we can get good generalization performance on the testing individuals irrespective of the number of SNPs that are given as input to the model (Supplementary Material, Figure S2B). This is an important point for a real-world application, as we could for instance have a number of different predictors and select the optimal one by comparing performance on a small number of individuals from the target population.

### Opportunities and future work

The penalized regression models that we consider in our study are based on the conventional oversimplifying assumption that the phenotypic observations are independently and identically distributed (i.i.d.) given the genotypes and the model parameters. In other words, we assume that the mapping (learned model parameters) from SNPs to the phenotype is homogeneous and identical for all individuals in the sampled population, and that for any individual a genetic prediction of their phenotype depends only on the learned mapping and that individual's genotype. When we have related and unrelated individuals in the training data, this assumption may not hold, as samples from the same family may exhibit internal dependencies. The i.i.d. assumption can also be limiting in other practical situations; e.g. if different genotypic markers or different mappings are predictive in different strata of the population. In the future, we will consider approaches that either explicitly model known internal dependencies—e.g. mixed-effects models with families fitted as random effects—or allow for non-homogeneous mappings to be learned from the data—e.g. by combining regression with a clustering method. Additional information may be needed in order to learn these more complex models, as the small sample size of our data sets poses a statistical limitation. For instance, information from multiple correlated traits could help infer potential sub-groupings when the source of internal dependencies is unknown.

Another interesting direction we plan to investigate in the future is the construction and evaluation of models that incorporate summary statistics from large GWAMAs in different ways. In this study, we showed that by combining dense whole-genome predictors with GWAMA-based polygenic scores in a simple meta-model, we achieve higher prediction accuracy than by using either model on its own. In light of this finding and given the increasing number of GWAMA studies becoming available through the organization of large consortia for different complex traits, we believe that comparing different ways of incorporating information from summary statistics into predictive models is an interesting research direction. For instance, we could use GWAMA summary statistics to define informative prior distributions for SNP effects, or to (re)-weight the SNP contributions, e.g. similar to the weighted G-BLUP model proposed in (25). Establishing the optimal way of combining external summary statistics with data statistics is of practical importance as it can lead to improved genomic predictors without the need to access large-scale individual-level data.

Furthermore, we believe that our meta-model is a simple, yet effective approach for combining predictors based on different data sources. Here, we only considered the combination of a dense, whole-genome predictor with the GWAMA-based risk score. Depending on which sources of data are available, other predictors and combinations could also lead to improved prediction accuracy, including predictors based on a recorded pedigree, and combinations of dense with sparse models estimated from the same or different data. For example, if we have data from different cohorts, we could consider combining a number of sparse predictors, each estimated using the data from a single cohort, and then learn the combination weights using a few individuals from the target cohort.

The GWAMA-based polygenic risk scores that we evaluated in this work are constructed using only the SNPs whose *P*-value of association passes the Bonferroni-corrected significance threshold in the GWAMA for each trait. However, as already discussed this is a stringent threshold and potentially leads to false negatives. We could therefore consider risk scores based on less-stringent thresholds. This is examined in (40) who find that using a less stringent threshold for the inclusion of SNPs to a polygenic risk score of schizophrenia results in a higher proportion of the variance in case–control status of testing individuals being explained. However, if we use GWAMA summary statistics and evaluate our risk scores in cohorts that have been used in the GWAMA study, then we need to be careful with the interpretation of our estimate of the prediction accuracy, especially if we are using denser risk scores resulting from applying a less-stringent threshold for selecting SNPs. The overestimation of prediction accuracy occurring when the testing individuals have been used in the marker selection step is examined in (26) and (41), who show that one of the important factors defining the amount of upward bias in the prediction accuracy is the number of SNPs included in the model.

Another important consideration when constructing GWAMA-based risk scores is how to deal with the LD structure. Selecting the SNPs based on univariate statistics can lead to redundancy in the selected SNPs, while using arbitrary cut-off thresholds can result in either uninformative data being used or useful information being ignored. An alternative to using the published summary statistics to directly construct a GWAMA score is to use them as priors for the amount of shrinkage applied to each SNP in penalized regression models. Incorporating GWAMA summary statistics as priors in penalized regression models could remove the need to account for LD structure, since in these models the effects of all SNPs are estimated jointly, while taking advantage of the predictive information coming from the large sample size of GWAMA studies by applying differential shrinkage to the input SNPs.

Finally, an important limitation of genomic predictors comes from the heritability of the trait (26). By definition, a complex trait is influenced by many factors, both genetic and environmental. Therefore, the proportion of trait variation that is due to genetic factors will always limit the accuracy we can achieve with a genetic predictor. The genomic predictors we developed perform statistically better than simply predicting the mean phenotype in many scenarios, certifying that there is an underlying predictive signal in genomic data. Therefore, identifying how we can use the predictive signal from genomic data as leverage into existing clinical models may lead to faster implementation of genomic findings in medical decision-making for complex traits. In the future, we will investigate how we can improve prediction accuracy in complex traits by combining genomic data with intermediate phenotypes, such as gene expression and methylation, similar to the OmicKriging method developed by Wheeler *et al*. (42). Such intermediate phenotypes can mediate the predictive signal from the genomic data, but also capture a proportion of the phenotypic variation that is due to current or past environmental influences.

## Conclusion

In this work, we focused on genetic predictors of complex traits and assessed performance under different scenarios with respect to trait architecture and population structure, mainly in terms of relatedness. The main motivation for considering genetic predictors is their potential benefit to clinical decision-making as implemented in a ‘personalized genomic medicine’ scenario. Although the genomic predictors we developed perform statistically better than chance in many scenarios—certifying that there is an underlying predictive signal in genomic data—the predictive accuracy of our models is still low for clinical decision-making at the level of the individual. However, the creation of national biobanks potentially linked with medical data from general practice, will open up new opportunities for genomic prediction, as having related individuals with recorded genotypic and phenotypic information will be likely for a large proportion of the population. The results from the meta-model we developed demonstrate that by combining dense whole-genome predictors with GWAMA-based risk scores we can improve prediction accuracy. Hence genomic predictors can be improved using summary statistics and without requiring access to large individual-level data sets, which are often difficult to share due to practical, legal and ethical considerations. The meta-model is a way of performing predictive meta-analysis for optimizing genomic predictions, and we believe that it can be extended to combine multiple information sources, potentially involving non-SNP data.

## Materials and Methods

### Data set description and pre-processing

In this work, we consider two data sets comprising measured phenotypes and genotypes of individuals from two European countries, Croatia (South-East Europe) and Scotland (North-West Europe). The first data set comprises individuals recruited in three locations in Croatia, the Dalmatian islands of Vis (43) and Korcula (44) and the mainland city of Split (45). We refer to this data set as ‘Croatia’. The second data set comprises individuals recruited in the isolated Scottish islands of Orkney as part of the Orkney Complex Disease Study (ORCADES) (46) and we refer to it as ‘Orkney’. In all cohort studies individuals were recruited irrespective of any specific phenotype. However, in Orkney and to a lesser extent in Vis and Korcula emphasis was given in recruiting family members of existing participants.

### Phenotypes

The traits we examine are height, body mass index (BMI) and high-density lipoprotein (HDL) cholesterol level. After removing individuals with extreme phenotypic measurements (values more than three standard deviations away from the population mean), the number of samples that have measurements for all three traits are 2186 in Croatia (Korcula = 816, Vis = 897, Split = 473) and 831 in Orkney. We log-transform the raw HDL measurements, in order to have phenotypic values that are normally distributed. Furthermore, we adjust each trait for the effects of sex, age and age squared within each cohort using a linear, additive model. Finally, we standardize the residuals from this analysis within each cohort. Overall, the phenotypes we consider in our prediction models are standardized residuals of the (log-transformed for HDL) measurements for each trait. We use only the training data to estimate the parameters of the linear model and the population mean and standard deviation that are subsequently used to adjust and standardize all data samples, unless explicitly stated in the text.

In principle, we can include covariates such as sex and age as inputs into the prediction models, and estimate the effects of these covariates and of the SNPs simultaneously. There are several practical advantages in pre-adjusting for these covariates. First, we can directly assess the prediction accuracy due to the genetic component, a correlation higher than zero (or a mean squared error smaller than one in the case of a standardized phenotype) suggests that the SNPs are informative of the trait. If we include the covariates as inputs then we need to compare performance over a baseline model that only considers the covariates, in order to assess any improvement in prediction accuracy attributable to the SNPs. Second, pre-adjusting for the covariates allows us to take into account more complex dependencies among these covariates, especially when we have multiple cohorts. We know from epidemiological studies that the effects of such covariates can be different in different populations. If we want to fit a model using samples from multiple populations, we therefore need to fit not only the covariates, but also possible interaction effects between the covariates and a population index. This is particularly relevant when we analyse the Croatian cohort, which comprises samples from three sub-populations in Croatia. Finally, using pre-adjusted phenotypes can facilitate comparison with models that do not model inputs in the same way as a linear parametric model, e.g. kernel methods employing non-linear kernel constructions. Potential disadvantages of pre-adjusting the phenotypes for covariates include the difficulty in interpreting how the predicted values relate to the true phenotypic scale, and the possible misspecification of our models if there are interactions between the pre-adjusted covariates and the genetic effects.

### Genotypes

All populations were genotyped using Illumina common SNP arrays. Vis was genotyped using the Illumina Infinium HumanHap300 BeadChip. The majority of Orkney samples were also genotyped using this chip with the remainder genotyped using the Illumina HumanHap370CNV duo chip. Korcula and Split were genotyped on the Illumina HumanHap370CNV duo and quad chips, respectively. We performed quality control in each cohort, excluding individuals with a call rate below 97% and SNPs with a call rate below 98%, or with a MAF of <1%, or when the *P*-value for deviation from Hardy–Weinberg equilibrium is smaller than 1 × 10^−6^. Following these quality control steps, we have 267 912 SNPs that are genotyped in all the Croatian sub-populations and 260 562 of them are also genotyped in Orkney. When we estimate the genomic predictors using our data sets, we standardize the genotypes using the frequency of the minor allele. Let *p_j_* be the MAF of SNP *j*, and $x_{ij}\in \{0,1,2\}$ denote the counts of the minor allele at SNP *j* for some individual *i*. Then the MAF-adjusted genotype is given by $\hat{x_{ij}}=(x_{ij}-2\;p_{j})/\sqrt{2\;p_{j}(1-\;p_{j})}.$ We note that with penalized regression methods such as LASSO and RR, the coding of the genotypes has an impact on model performance, as the penalty applied to each coefficient depends on the scale of the covariate. By standardizing the genotypes, we effectively impose the same penalty on all the SNPs. In experiments that use samples from both Croatia and Orkney, we do not use the 7350 SNPs that are not genotyped in Orkney. For the GWAMA-score construction, SNPs that are not genotyped (i.e. not included in the 267 912 SNPs) are imputed based on the 1000 Genomes data set. Imputation of ∼39 million SNPs and insertions/deletions was competed for Vis, Korcula, Split and ORCADES using the ‘ALL (Phase 1 integrated release v3, April 2012)’ reference panel. SHAPEIT (47) version 2 was used for pre-phasing because it can partially utilize pedigrees to improve the phasing for related individuals. Imputation was completed using IMPUTE2 (48).

### Ethics statement

All the Croatian cohorts, referred to as Vis, Korcula and Split, received ethical approval from the Ethics Committee of the Medical School, University of Split and the NHS Lothian (South East Scotland Research Ethics Committee). The ORCADES study, referred to as Orkney, received ethical approval from the NHS Orkney Research Ethics Committee and North of Scotland Research Ethics Committee. All studies followed the tenets of the 1975 Declaration of Helsinki and all participants gave written informed consent prior to participation.

### General prediction setting

The common case in genomic predictions of complex traits is to have a data set *D* comprising *n* individuals, whose genotypes at a number of loci are measured along with the phenotypic value of interest. Measurements for additional covariates, such as age and sex, are often available. We will refer to such data measurements as ‘side information’. These are not directly of interest, but can be used as covariates in the model—in which case the resulting predictor is not solely based on genomic information—or they can be used to ‘adjust’ the phenotype prior to building a predictive model—in which case we want our model to capture the genomic signal that is predictive of the phenotype of interest and disregard any genomic signal that is predictive of the side information.

We will denote this data set by $D=\{y_{i},{\vec{x}}_{i},{\vec{z}}_{i}|i=1\ldots n\},$ where *y_i_* is the phenotype of individual *i*, **x***_i_* is a vector of genotypes for individual *i*, with $x_{ij}\in \{0,1,2\}$ denoting the counts of a specific allele at SNP *j*, and we assume that all markers are diallelic, and **z***_i_* is a vector with measurements of side information for individual *i*. Note that we consider a univariate phenotype of interest, i.e. *y_i_* is scalar. Given such a data set *D*, our aim is to construct a predictive model, which given the genotypes of a new individual, x_*_, and any side information, z_*_ (if our predictor is not solely genomic), can give us a prediction about the unobserved phenotype, y_*_. In this work, we consider quantitative traits with continuous phenotypes, hence the prediction task can be specified as a regression problem. To this end, the statistical models used in quantitative genetics to describe the variation of a phenotypic trait are of the following form (16):

(1) $$y_{i}=f(x_{i},z_{i}|\theta )+\epsilon_{i},\;\epsilon_{i}∼N(0,\sigma_{\epsilon }^{2}),$$

where $f({\vec{x}}_{i},{\vec{z}}_{i}|\theta )$ is a function modelling the effects of the genetic and side information on the phenotype, ***θ*** is a vector of parameters that we typically want to estimate from our data, and *ϵ* is a model residual, assumed to be independently and identically distributed (i.i.d.) from a normal distribution, with mean 0 and variance $\sigma_{\epsilon }^{2}.$ In quantitative genetics, *ϵ* is assumed to include all sources of phenotypic variation that are omitted from $f({\vec{x}}_{i},{\vec{z}}_{i}|\theta )$ and it is often referred to as the ‘environmental component’ of a phenotypic trait, i.e. some random source which is independent from the ‘genetic component’ and side information modelled through $f(x_{i},z_{i}|\theta )$.

In this work, we pre-adjust the phenotypes with respect to side information, so that *y_i_* is a standardized residual, resulting from adjusting the original phenotype for the effect of sex, age and age squared and subsequently centring and scaling to (0, 1). Therefore, our statistical model of the phenotype can be simplified as:

(2) $$y_{i}=g(x_{i}|\beta )+\epsilon_{i},\;\epsilon_{i}∼N(0,\sigma_{\epsilon }^{2}),$$

where $g({\vec{x}}_{i}|\beta )$ is a function modelling genetic effects on the phenotype. We note that the residual variance, $\sigma_{\epsilon }^{2},$ is different to that appearing in Equation (1), but we have used the same notation for simplicity. Furthermore, we only consider a linear, additive model for this ‘genetic component’ of phenotypic variation, with

(3) $$g(x_{i}|\beta )=\sum_{\;j=1}^{P}\beta_{j}x_{ij},$$

where *P* is the number of SNPs that we consider, and *β_j_* is the partial regression of the phenotype on SNP *j*.

There are several ways of selecting the genomic markers that enter Equation (3) and of estimating the parameter vector *β*. Here, we consider two methodologically distinct approaches; a GWAMA-based polygenic score, which is entirely based on results from the literature, and a range of penalized regression models with and without pre-filtering of markers, which are entirely based on estimation procedures using our own data.

### GWAMA-based polygenic score

This score is constructed using results from large GWAMA that evaluate the univariate association of each marker (SNP) with the phenotype based on more than 100 000 individuals and often exceeding 200 000. For each trait, we only use the SNPs that are reported as significant associations in the corresponding GWAMA article. We construct the polygenic score for each individual by adding the number of reference alleles he or she carries at the selected SNPs, pre-multiplied by the corresponding SNP effect sizes reported in the GWAMA article.

More specifically, to construct the GWAMA-based polygenic score for height, we use the 180 SNPs identified by the GIANT Consortium and reported in Supplementary Material, Table S1 of (33), together with the reported effect sizes (Beta from Stage 1 + Stage 2, in the aforementioned table). Similarly, for the BMI GWAMA-based polygenic score we use the 32 SNPs reported in Table 1 of (34), with effect sizes taken from the ‘per allele change in BMI’ column. Finally, for the HLD GWAMA-based polygenic score, we use 70 SNPs reported as associated with HDL in Tables 1–4 and Supplementary Material, Table S3 of (35), together with the reported effect sizes that correspond to HDL.

When the reported SNPs are not included in our genotyping array, we use allelic dosages—imputed using the 1000 Genomes reference data and the existing genotype data—to construct the GWAMA scores. In fact, <30% of the reported SNP ‘hits’ for each trait are genotyped in our data (22 out of 180 in height, 7 out of 32 in BMI and 20 out of 70 in HDL). However, imputed dosages with relatively good imputation quality are available for the non-genotyped SNP ‘hits’ and used for the GWAMA score construction, with the exception of three height SNP ‘hits’ whose position does not exist in the genome build of the 1000 Genomes data set used to perform the imputation.

A list of the GWAMA ‘hits’ for each trait, together with information on genotyping status, imputation quality and the GWAMA estimated beta coefficients that we used to construct the scores is given in Supplementary Material, Table S2.

We note that the Croatia and the Orkney cohorts are used in the GWAMA studies of all three traits, and hence the prediction accuracy of the GWAMA score will have an upward bias (26). Given that the number of individuals used to estimate the GWAMA effects is large (>100 000) and that we only use a small number of SNPs (180, 32, 70 for height, BMI and HDL, respectively) this bias will be small.

### Penalized regression methods

We examine two of the most commonly used shrinkage estimation methods, namely RR (30)—equivalent to the (Genome-enabled) Best Linear Unbiased Predictor, (G)-BLUP (19), which is widely used in animal and plant breeding—and the Least Absolute Shrinkage and Selection Operator (LASSO) (31). We also examine a combination of these two estimation methods termed the Elastic Net (EN) (32). The Least-Squares solution for the model defined by Equations (2) and (3) is given by

(4) $$arg_{\beta }min\sum_{i=1}^{n}{\left(y_{i}-\sum_{\;j=1}^{P}\beta_{j}x_{ij}\right)}^{2},$$

i.e. to get the best fit for data set *D*, we adjust the values of the parameter vector *β* so as to minimize the sum of the squared terms of model residuals.

The solution in Equation (4) minimizes the prediction error in data set *D*. However, what we are interested in is whether a model has good predictive performance in *new, unseen* data points, i.e. we are interested in whether a model *generalizes* well*.* In penalized regression models, generalization is achieved by introducing a regularization (penalty) term to the least squares objective, which pushes the resulting model towards a smoother underlying function, by shrinking the model parameters towards 0. The implicit assumption is that the underlying process that generates the data is ‘smoother’ than what the data actually suggests, and thus the penalty term prevents the model from fitting the *noise* in the data, a problem also referred to as *over-fitting*. The objective function now has two components

(5) $$arg_{\beta }min\sum_{i=1}^{n}{\left(y_{i}-\sum_{\;j=1}^{P}\beta_{j}x_{ij}\right)}^{2}+\lambda r(\beta ),$$

where r(β) is the penalty term and *λ* is a meta-parameter controlling the amount of regularization. The penalty term introduces some bias to the least-squares solution, preventing the *β_j_*'s from fitting the data points in *D* perfectly. However, too much regularization will also lead to ignoring the signal in the data, and thus the optimal *λ* should be chosen according to a validation set, as discussed in the section *Experimental procedure and evaluation*. Assigning a prior distribution over the model parameters and inferring quantities of interest by computing their posterior distribution, can also achieve regularization.

The three methods we examine have different penalty terms. RR has an $\ell_{2}$ penalty term, $r(\beta )=\sum_{\;j=1}^{P}\beta_{j}^{2},$ which results in a *dense* model, meaning that all the *P* markers are assigned a non-zero weight, albeit shrunk. LASSO has an $\ell_{1}$ penalty term, $r(\beta )=\sum_{\;j=1}^{P}|\beta_{j}|,$ which results in a *sparse* model, meaning that a lot of markers are given a 0 weight, and thus no longer contribute to the predictive model. A sparse solution can be advantageous, as it is more interpretable and easier to implement—we need to genotype fewer markers to make predictions in new individuals. However, the LASSO penalty tends to select only one variable from a group of correlated variables and could therefore prune markers that carry additional information if they are in high LD with markers already included in the solution. The EN combines the RR and LASSO penalties, and overcomes this limitation, while still leading to a sparse solution when compared with RR. The EN penalty term is $r(\beta )=\alpha \sum_{\;j=1}^{P}\beta_{j}^{2}+(1-\alpha )\sum_{\;j=1}^{P}|\beta_{j}|$, where *α* is a second meta-parameter controlling the proportion of $\ell_{1}$ to $\ell_{2}$ penalty. We refer the reader to (19) for a broader overview of shrinkage estimation methods, both point estimates and Bayesian methods, that have been used in the context of genomic predictions to infer the parameters of the model defined by Equations (2) and (3). Interestingly, the LASSO and the RR solutions can be derived as the maximum-a-posteriori estimates when placing a Laplace and a Gaussian distribution as prior over the model parameters, respectively.

Finally, we note that the problem of over-fitting, and hence the need for regularization, is particularly prominent when we want to fit a complex model with relatively few data points (here, individuals). In the context of genomic predictions, this problem is relevant when we consider whole-genome regression methods. This is often referred to as the ‘large p small n’ situation, where the number of model parameters, *β_j_*'s, corresponds to the number of genomic markers, *P*, and is typically one or two orders of magnitude larger than the number of samples (individuals), *n*, in a data set. When *P* >> *n*, the solution to the least squares objective in Equation (4) will fit the data points in *D* perfectly. However, a lot of the *β_j_*'s will be describing the ‘noise’ in *D*, i.e. unique idiosyncrasies of the specific data set, which do not generalize in the overall population.

### GWAS-based pre-filtering of SNPs

A different approach for addressing the problem of over-fitting is to pre-filter the genomic markers, that is, to select in advance the markers to be used as explanatory variables in the regression. This can be done by ranking the markers according to some usefulness criterion and using only the top *m* markers in the prediction model defined by Equation (2). This pre-selection step is often termed as ‘feature selection’ and is an important step in most applications of statistical learning.

Here, we pre-select markers by computing the association between each SNP and a trait and selecting the *m* SNPs with the lowest *P*-values of association, where m∈{5,10,100,500,1k,10k,25k,100k,200k} and *k* denotes thousands. The *P*-value of association is computed using the full training data set in each scenario, with the following exception where only a subset of the training data set is used. In the experiment where we add the Croatian samples to the training folds of the within-cohort nested cross-validation procedure in Orkney, we use the *P*-values estimated using the Orkney training data only. In this case, the Croatian samples are only used during the training of the penalized regression models and not during the GWAS pre-filtering.

This GWAS-based pre-filtering does not take LD into account, i.e. it selects the *m* most associated SNPs, rather than the *m* most associated *independent* loci. However, this pre-filtering procedure is simple to implement—it does not require characterization of the LD structure, which can be computationally expensive, or evaluation of different LD thresholds—while the potential redundancies in the selected SNPs can be dealt with by the penalized regression methods, which estimate the SNP effects jointly, and tend to shrink the parameters of correlated variables that do not contribute complementary predictive signal.

### Meta-models

In statistical learning, we can often achieve better predictive performance by combining the predictions from multiple models. This is often called model averaging (49) or ensemble learning (50,51) and the main idea is that each model in the ensemble makes different assumptions about the underlying mappings and possibly explains only a part of the data structure. By combining multiple models in a flexible way, we can produce a stronger model that explains more of the data structure.

Here, we construct such an ensemble by combining the GWAMA-based polygenic score with the best shrinkage model estimated using our data sets. The main motivation is to test if these two predictors combined can achieve higher prediction accuracy than either of the predictors on its own. If that were the case, it would mean that they capture different aspects of the data structure that is predictive of a phenotype.

More specifically, let us consider a single trait, and let $\hat{y_{i|S}}$ denote the polygenic score for individual *i*, and $\hat{y_{i|R}}$ denote the predicted value for individual *i* under the best penalized regression model, where the optimal model is selected using an independent, validation data set. Then, a simple meta-model that combines these two predictors is given by

(6) $$y_{i}=\beta_{S}\hat{y_{i|S}}+\beta_{R}\hat{y_{i|R}}+\epsilon_{i},\;\epsilon_{i}∼N(0,\sigma_{\epsilon }^{2}),$$

where *β_S_* and *β_R_* are parameters of the meta-model that we need to estimate from data, and *Y_i_* is the phenotype of individual *i*. Again, the residual variance, $\sigma_{\epsilon }^{2},$ is different to that appearing in Equation (1) and in Equation (2), but we have used the same notation for simplicity. We estimate *β_S_* and *β_R_* using ordinary least squares. We discuss the data partition scheme for training and evaluating the meta-model in the section *Experimental procedure and evaluation*.

### Model comparison

To compare model performance, we apply the Fisher *z*-transformation on the correlation coefficient corresponding to each model. Given *z_M_*_1_ and *z_M_*_2_ for models *M*_1_ and *M*_2_, we test if model *M*_1_ is better than model *M*_2_ by performing a one-tailed paired *z*-test. Statistical significance is evaluated at the 0.05 *P*-value threshold.

### Experimental procedure and evaluation

In order to get an unbiased estimate of prediction accuracy in new unseen individuals, we need two mutually exclusive data sets: a training set which we use to perform feature selection and to estimate model parameters, and a testing set which we use to estimate the prediction accuracy of our predictive model. A third data set is additionally needed when we want to perform *model selection*, i.e. when we consider a range of candidate predictive models and want to select the best among them for deployment. This data set is called the validation set and is used to compare the performance of candidate models or model settings. It is important to note that in the validation step, our choice of the *optimal model* could be influenced by randomness in the data set [see, e.g. (52) for an application of bagging in GBLUP]. Therefore, if all candidate models and settings are equally likely a priori, then the accuracy of the optimal model on the validation set describes the *best-case scenario*, rather than the *most likely scenario*. To get an unbiased estimate of model performance in new unseen data, we need to measure performance on a data set that has not been used for any decision-making.

Furthermore, as pointed out in (26), to get an estimate of the prediction accuracy that we can expect in a target population, we need to perform this estimation using samples from that target population. Here we consider two scenarios, *within-cohort prediction*, in which case the training, validation and testing sets come from the same population cohort, and *across-cohort prediction*, in which case the training and testing sets come from different populations and the validation set comprises samples either from the training or from the testing population. In each scenario, we assume that the target population is the same as the testing population and examine how well different models generalize for different target populations and what are the characteristics of the trait that make such generalization possible.

In within-cohort prediction, we use nested cross-validation to split the data into training, validation and testing sets. In the ‘outer’ cross-validation phase, we split the data in 10 mutually exclusive folds, so that each 10% of the data samples is a separate testing set. In the ‘inner’ cross-validation phase, the remaining 90% of the data in each fold is further split into 5-folds for the purpose of training and validation; each training set comprises 72% of the data samples and each validation set comprises 18% of the data samples. Once model selection is performed, we join the validation and training samples and re-estimate the parameters of the optimal model using the full 90% of the data samples. Similar to (22), we perform model selection by running the inner cross-validation on a single randomly selected outer fold. This reduces the computational burden from 5 × 10 = 50 model computations to 5 + 10 = 15 model computations, and is found to be sufficient for model selection in similar work (22). A pictorial representation of the nested cross-validation procedure is given in Supplementary Material, Figure S4.

In across-cohort prediction, the training and testing samples are independent by construction, as they come from different populations. For the validation set, we consider two scenarios. First, the validation set comprises individuals from the training population. In this case, we use the results from the ‘outer’ 10-fold cross-validation to select the optimal model and then use the full training population data set to estimate the parameters of this optimal model and compute the prediction accuracy on the testing data (Supplementary Material, Fig. S5). In the second scenario, the validation set comprises individuals from the target (testing) population. In this case, we use a 10-fold split of the target population samples and use 90% to select the optimal model (validation set) and the remaining 10% to assess the prediction accuracy (testing set). The models are trained using the full training population data set (Supplementary Material, Fig. S6).

Finally, to estimate the parameters of the meta-models, we further split each testing fold from within-cohort prediction in 10 sub-folds and perform a cross-validation experiment to train the meta-model and assess its performance. The same testing samples are used to evaluate the performance of the single models and of the meta-models, but in the meta-models an additional data split is applied to perform the meta-training. Specifically, each testing fold (10% of the full data set) is split in meta-training and meta-testing samples, where the meta-training set comprises 9% of the full data set and the meta-testing set comprises 1% of the full data set (Supplementary Material, Fig. S7). This gives rise to a 100-fold cross-validation procedure: 10 testing folds, each split in a further 10 meta-folds, and is equivalent to a doubly nested cross-validation procedure, where 1% of the data is left-out for evaluating the accuracy of the meta-model and the remaining 99% is used for training, validation and testing purposes. We note that 9% of the data is a small sample, but the meta-model only has two parameters, and hence estimation is possible.

The optimal penalty strength, *λ*, for the shrinkage models is selected using grid search. For LASSO and the EN, we consider λ∈{0.0025,0.005,0.0075,0.01,0.025,0.05,0.075,0.1,0.25}×n, and for RR, we consider λ∈{0.01,0.05,0.1,0.5,1,10,50,100,250,500,1000,1500}×n, where *n* is the number of samples in the training set. To select the range of *λ* values for each model, we performed preliminary experiments using 500 randomly selected samples from the Croatian cohort. We evaluated performance using *λ* values with different orders of magnitude and expanded the range of values when performance was optimal (or close to the optimal) at either end of the spectrum. A more automated way of suggesting a good sequence of *λ* values is described in (53). The main idea is to compute the smallest *λ* value, *λ*_max_, for which all coefficients are shrunk to zero, select a minimum *λ* value, *λ*_min_, and evaluate a sequence of *K* values for *λ* decreasing from *λ*_max_ to *λ*_min_ on the log scale. The suggested values for *λ*_min_ and *K* are *λ*_min_ = 0.001 × *λ*_max_ and *K* = 100. The meta-parameter *α* that controls the proportion of $\ell_{1}$ to $\ell_{2}$ penalty in EN is optimized using grid search over α∈{0.2,0.4,0.6,0.8}. In principle, the EN can attain the LASSO and the RR solutions as special cases. However, here we constrain the EN solution to have non-zero contributions from both the $\ell_{1}$ and the $\ell_{2}$ penalty terms, since we are already evaluating the performance of RR and LASSO separately.

Throughout this article, we measure prediction accuracy using the Pearson's correlation coefficient between the true and the predicted phenotypes of the testing samples. When we use cross-validation, the predictions of the testing data from all the folds are jointly considered to compute the correlation. Note that although we do not report prediction accuracy on the validation set, this is used to select the meta-parameters *λ* and *α* in the shrinkage models, as well as the optimal number of input SNPs and the shrinkage type (denoted by the black ‘x’ symbol in Fig. 2).

## Supplementary Material

Supplementary Material is available at *HMG* online.

## Funding

This work was supported by the Biotechnology and Biological Sciences Research Council, UK (research grant number BB/I014144/1). We further acknowledge funding from the Medical Research Council, UK. Pharmatics acknowledges the support of European Commission Framework Programme 7 MIMOmics (FP7-HEALTH-305280). The CROATIA-Korcula study was funded by grants from the Medical Research Council (UK), European Commission Framework 6 project EUROSPAN (Contract no. LSHG-CT-2006-018947) and Republic of Croatia Ministry of Science, Education and Sports research grants to I.R. (108-1080315-0302). The CROATIA-Split study is funded by grants from the Medical Research Council (UK), European Commission Framework 6 project EUROSPAN (Contract no. LSHG-CT-2006-018947) and Republic of Croatia Ministry of Science, Education and Sports research grants to I.R. (108-1080315-0302). The CROATIA-Vis study was funded by grants from the Medical Research Council (UK) and Republic of Croatia Ministry of Science, Education and Sports research grants to I.R. (108-1080315-0302). ORCADES was supported by the Chief Scientist Office of the Scottish Government, the Royal Society, the MRC Human Genetics Unit, Arthritis Research UK and the European Union framework program 6 EUROSPAN project (Contract no. LSHG-CT-2006-018947). The funders had no role in study design, data collection and analysis, decision to publish or preparation of the manuscript. Funding to pay the Open Access publication charges for this article was provided by the Biotechnology and Biological Sciences Research Council (grant number BB/I014144/1).

## Supplementary Material

Supplementary Data
