# Supplementary material for: Genomic prediction of complex human traits: relatedness, trait architecture and predictive meta-models
Source: Hum Mol Genet. 2015 Apr 26;24(14):4167–82. doi: 10.1093/hmg/ddv145 (PMC4476450; doi:10.1093/hmg/ddv145)
Supplement: Supplementary Data [file supp_24_14_4167__index.html]

Genomic prediction of complex human traits: relatedness, trait architecture and predictive meta-models — Supplementary Data 

# Genomic prediction of complex human traits: relatedness, trait architecture and predictive meta-models

## Supplementary Data

Supplementary Data

- Supplementary data\_Figures - pdf file
- Supplementary Table 1 - pdf file
- Supplementary Table 2 - xls file
