# Supplementary material for: Genomic prediction of complex human traits: relatedness, trait architecture and predictive meta-models
Source: Hum Mol Genet. 2015 Apr 26;24(14):4167–82. doi: 10.1093/hmg/ddv145 (PMC4476450; doi:10.1093/hmg/ddv145)
Supplement: Supplementary Data [file supp_ddv145_ddv145supp_data_figs.pdf]

# Supplementary Information:
# 'Genomic prediction of complex human traits: relatedness, trait architecture, and predictive meta-models'

## List of Figures

S1 Histograms of GWAMA-estimated SNP effect sizes for Height, BMI and HDL . . . . . . . 2
S2 Accuracy of penalized regression models and GWAMA scores under different scenarios. . 3
S3 Relatedness between training and testing samples from different scenarios. . . . . . . . . . 4
S4 Data split in nested cross-validation procedure used in within-cohort prediction. . . . . . . 5
S5 Data split for scenario 1 in across-cohort prediction. . . . . . . . . . . . . . . . . . . . . . . . 6
S6 Data split for scenario 2 in across-cohort prediction. . . . . . . . . . . . . . . . . . . . . . . . 7
S7 Data split for training and evaluation of the meta-model. . . . . . . . . . . . . . . . . . . . 8
S8 Accuracy of LASSO, RR and GWAMA score using 'related', 'unrelated' and 'all' testing individuals based on IBS $\geq 0.0625$ . . . . . . . . . . . . . . . . . . . . . . . . . . . . . 9

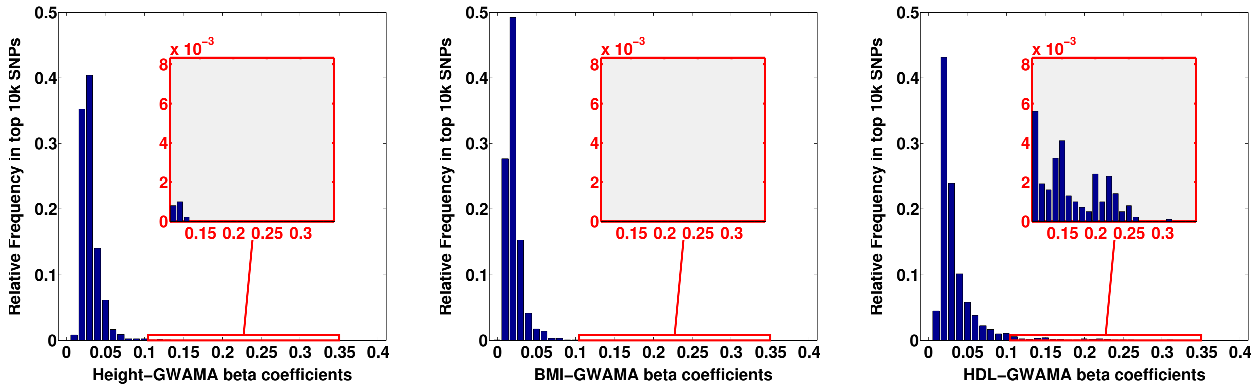


**Supplementary Figure S1.** Histograms of effect sizes (in standard deviation units) of the 10,000 most associated SNPs estimated in Genome-Wide Association Meta-Analyses (GWAMA) of Height, BMI and HDL. The gray sub-panels display in more detail the right tail of the histograms by zooming-in on the $y$-axis. The empirical distribution of SNP effect sizes in HDL has a fat tail, compared to that of height and of BMI, indicating some moderately-sized effects.

The beta coefficients are given in standard deviation units. For HDL we have used the results from the joint analysis of metabochip and GWAS data performed by (Global Lipids Genetics Consortium et al., 2013), and available from `http://www.sph.umich.edu/csg/abecasis/public/lipids2013/`. In this analysis, HDL was adjusted within each study cohort for age, age$^2$ and sex and the residuals were then quantile normalized. For Height and BMI we have used results from the GIANT Consortium sex stratified anthropometrics analyses performed by (Randall et al., 2013), and available from `http://www.broadinstitute.org/collaboration/giant/index.php/GIANT_consortium_data_files`. In these analyses, height and BMI were adjusted for age and age$^2$ for men and women separately. In BMI a rank-based inverse normal transformation of the residuals was used, whereas in height the residuals were normalized by taking a z-score transformation. For height and BMI, we plot the average of the beta coefficient estimated in men and in women. To select the top 10,000 SNPs, we have a two-step procedure. First, we select SNPs for which at least $N \geq 50,000$ individuals were analysed (2,337,961/2,324,092/2,447,441 SNPs in height, BMI and HDL respectively). Then, we select the 10,000 SNPs with the lowest p-value of association.

# References


Global Lipids Genetics Consortium, Willer, C. J., Schmidt, E. M., Sengupta, S., Peloso, G. M., et al. (2013). Discovery and refinement of loci associated with lipid levels. *Nature Genetics*, 45(11):1274–1283.

Randall, J. C., Winkler, T. W., Kutalik, Z., Berndt, S. I., Jackson, A. U., et al. (2013). Sex-stratified genome-wide association studies including 270,000 individuals show sexual dimorphism in genetic loci for anthropometric traits. *PLoS Genetics*, 9(6):e1003500.

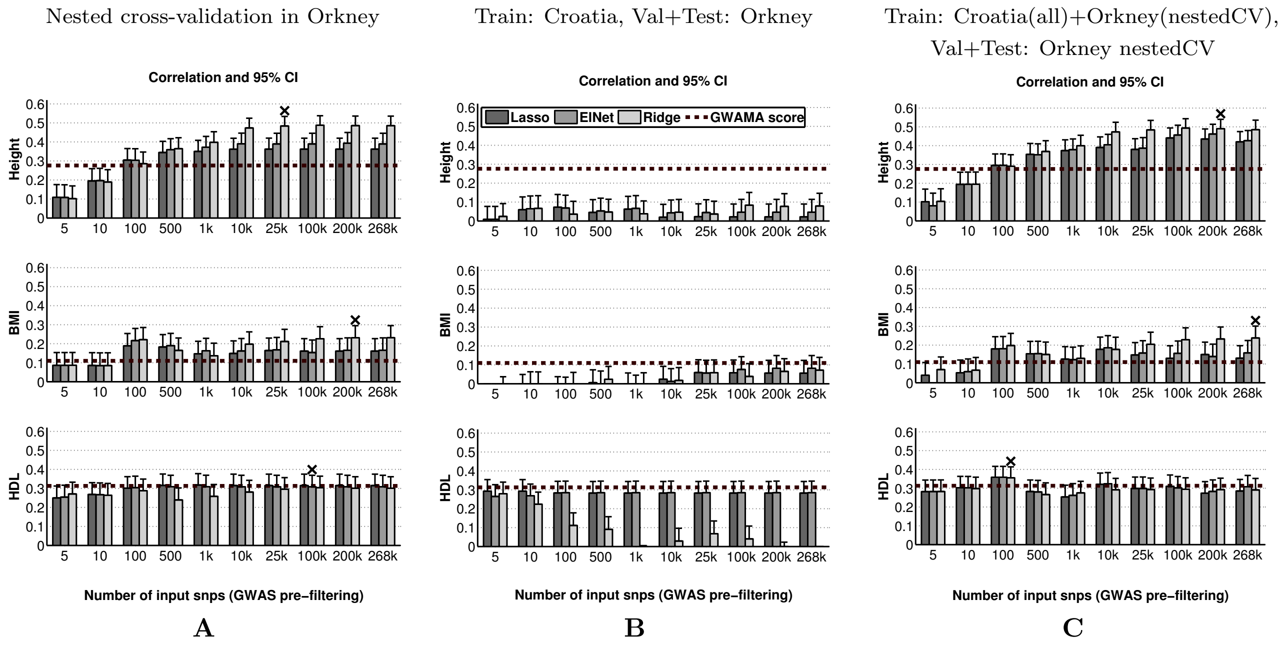


**Supplementary Figure S2.** Accuracy of penalized regression models as we increase the number of input SNPs, together with accuracy of the GWAMA-based polygenic score. (**A**) Within-cohort prediction in Orkney (repeated from Figure 2 to facilitate comparison). (**B**) Across-cohort prediction scenario 2 (training: Croatia, validation & testing: Orkney). (**C**) Prediction in Orkney using combined datasets (nested cross-validation in Orkney and Croatia samples added to all training folds). y-axis: accuracy is measured using the Pearson's correlation coefficient between the predicted and true phenotype of the testing samples. The error bar corresponds to the 95% Confidence Interval (upper CI). x-axis: an increasing number of SNPs are given as input to the regression models. The SNP selection is performed by GWAS pre-filtering using the training data in each case. The number of input SNPs for the GWAMA-based risk score is constant (height: 180 SNPs, BMI: 32 SNPs, HDL: 70 SNPs). *Black 'x' symbol*: depicts the optimal penalized regression model in terms of the type of shrinkage penalty and the number of input markers based on prediction accuracy on the validation set.

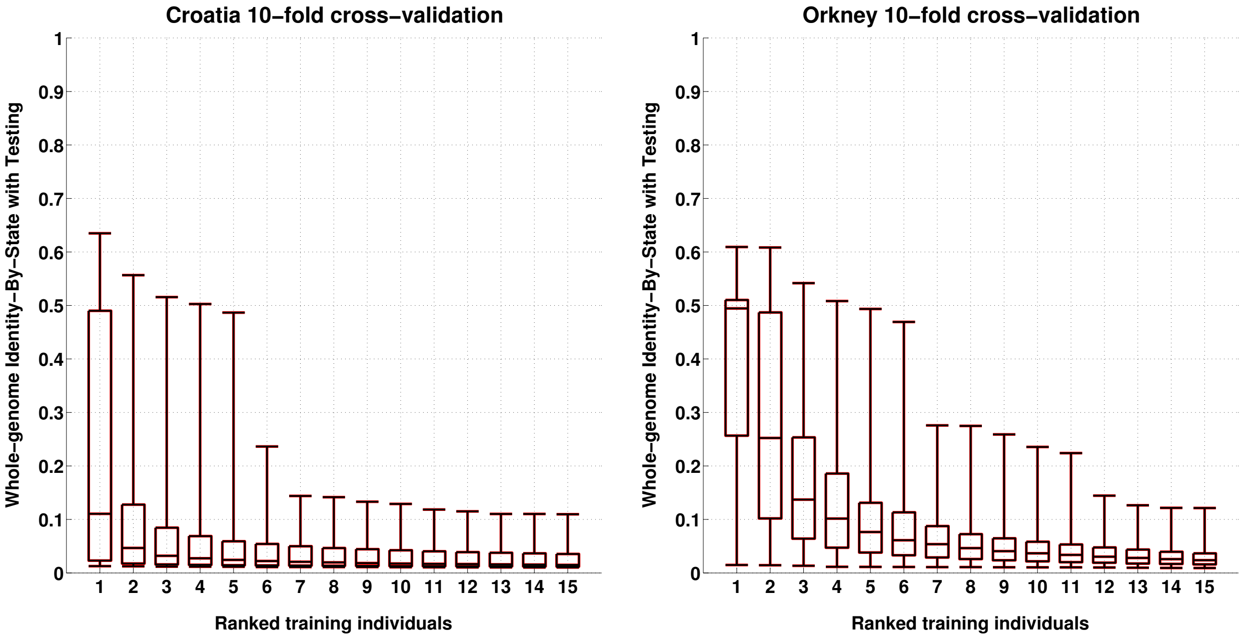


**Supplementary Figure S3.** Relatedness between training and testing samples in within-cohort prediction in Croatia (left) and in Orkney (right). Each panel shows boxplots of the 15 highest similarities between each testing sample and the training samples. The similarity between individuals $i$ and $k$ is computed by $s_{ik} = \frac{1}{P}\sum_{j=1}^{P} \frac{(x_{ij}-2p_j)(x_{kj}-2p_j)}{2p_j(1-p_j)}$ , where $p_j$ is the minor allele frequency of SNP $j$, $x_{ij} \in \{0, 1, 2\}$ is the genotype of individual $i$ at SNP $j$, and $P = 267,912$ $(260,562$ in Orkney) is the number of the genotyped SNPs. In the Croatian dataset the median similarity to the most closely related training sample is less than 0.125, whereas in Orkney it is just under 0.5 (expected similarity between full siblings or parent-offspring). In addition, in Orkney the median similarity to the second and third most closely related training samples is still relatively high ($\sim 0.25$ and $\sim 0.125$, respectively), which means that more than half of the testing samples are nominally related to at least two training samples and occasionally multiple ones.

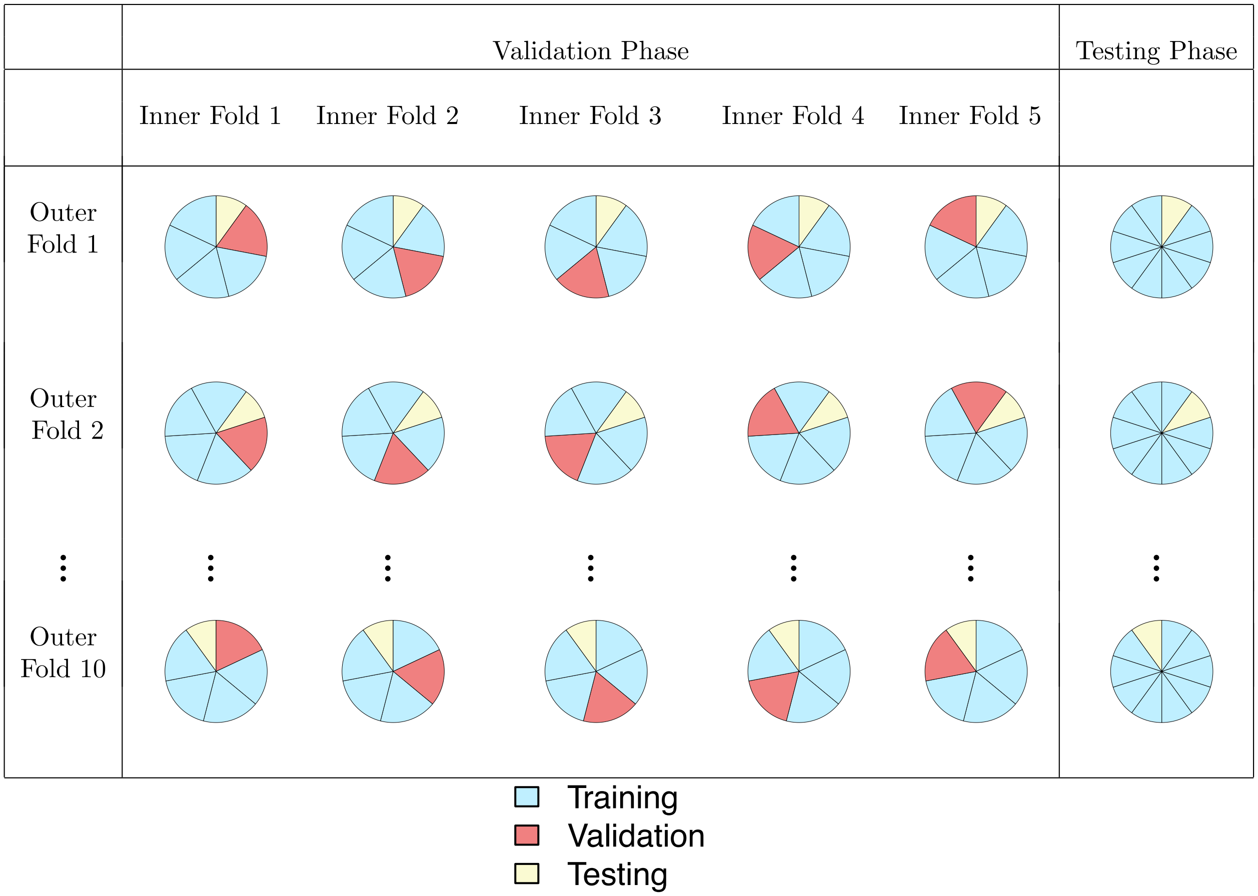


**Supplementary Figure S4.** Data split for the nested cross-validation procedure used in within-cohort prediction.
*During the validation phase:* for every outer fold we leave out the testing data (yellow part), and then for each inner fold we train the models using the light-blue part and make predictions on the red part. The accuracy in the validation set is estimated by joining the predictions from the red parts in each row. For each model, the penalty strength that achieves the highest accuracy in the validation set is taken forward to the *testing phase.* The overall model configuration – in terms of shrinkage type, number of input SNPs and penalty strength – that achieves the highest accuracy in the validation set is indicated by a black ‘x’ symbol in Figure 2 and Supplementary Figure 2 and is referred to as the ‘optimal’ penalized regression model in the text.
*During the testing phase (last column):* we apply the penalty strength selected in the validation phase and train the models using the light-blue part and make predictions on the yellow part. The prediction accuracy on the testing data is estimated by joining the predictions of the yellow parts in the last column, and is the one reported in the manuscript.

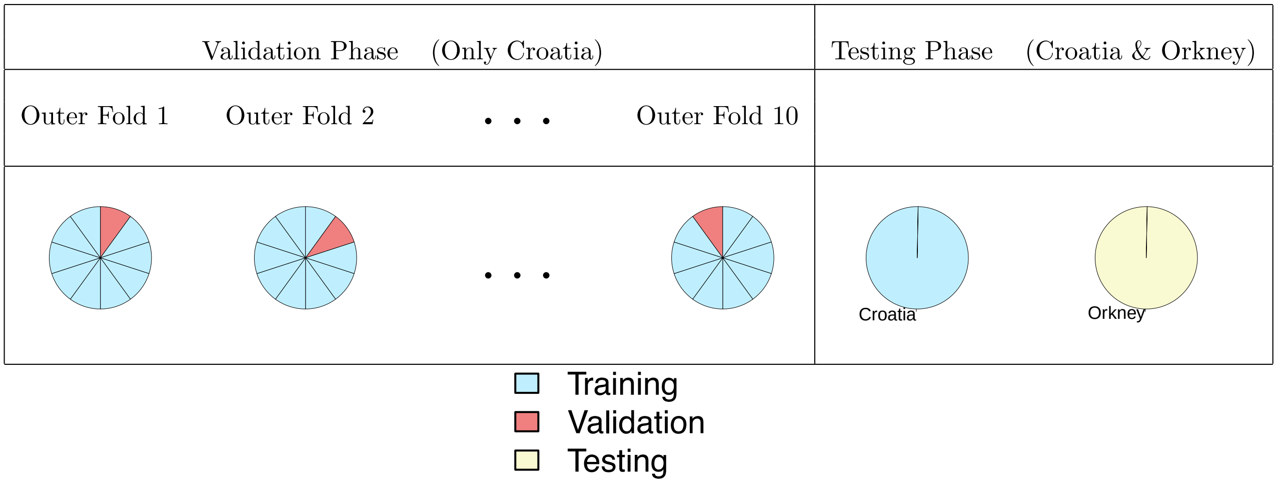


**Supplementary Figure S5.** Data split for scenario 1 in across-cohort prediction.
*During the validation phase:* we use a standard 10-fold cross-validation procedure with the samples from Croatia. For each model, the penalty strength that achieves the highest accuracy in the validation set is taken forward to the *testing phase*.
*During the testing phase:* we apply the penalty strength selected in the validation phase and train the models using all the individuals from Croatia (light-blue pie) and make predictions to all the individuals from Orkney (yellow pie).

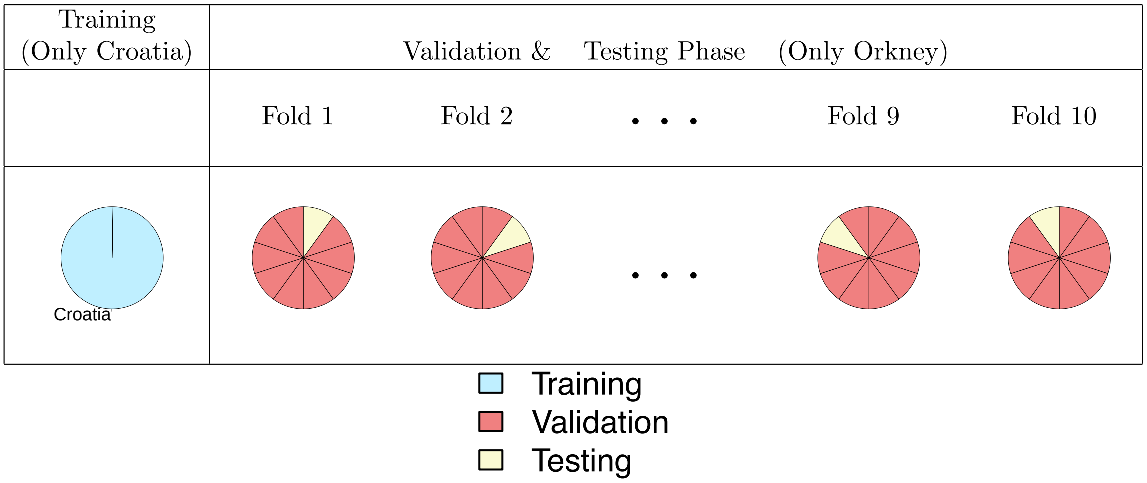


**Supplementary Figure S6.** Data split for scenario 2 in across-cohort prediction.
We perform training of all the models (with all the penalty strengths on the grid) using the Croatia samples. Then for each fold, we use the prediction accuracy estimated from the validation samples (red parts) to select the optimal penalty strength for each model, and using the model trained with this optimal penalty strength we make predictions on the held-out testing samples (yellow parts). The prediction accuracy on the testing data is estimated by joining the predictions of the yellow parts across folds, and is the one reported in the manuscript.

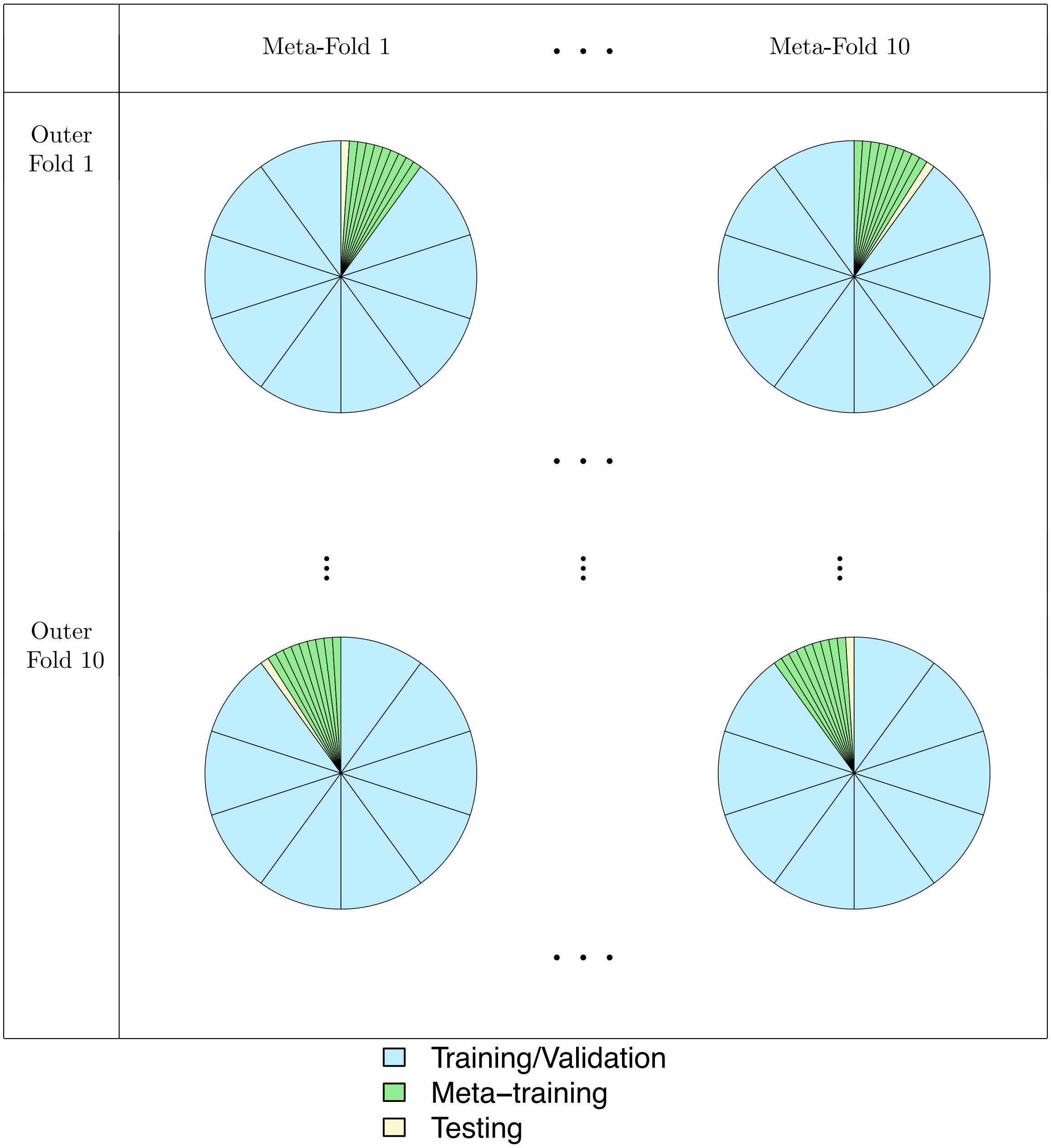


**Supplementary Figure S7.** Data split for training and evaluation of the meta-model.
For each outer fold we use the ‘training/validation’ data (light-blue parts) to train the penalized regression models and select the best penalty strength, number of input SNPs and shrinkage type (as in Supplementary Figure 4).
Then for each outer fold, we iteratively use 9/10 of the held-out data to perform training of the meta-model (green parts) and make predictions from the meta-model in the remaining 1/10 of the data (yellow part). The prediction accuracy on the testing data is estimated by joining the predictions of the yellow parts across all outer-folds and all meta-folds (100 small yellow pieces), and is the one reported in the manuscript.

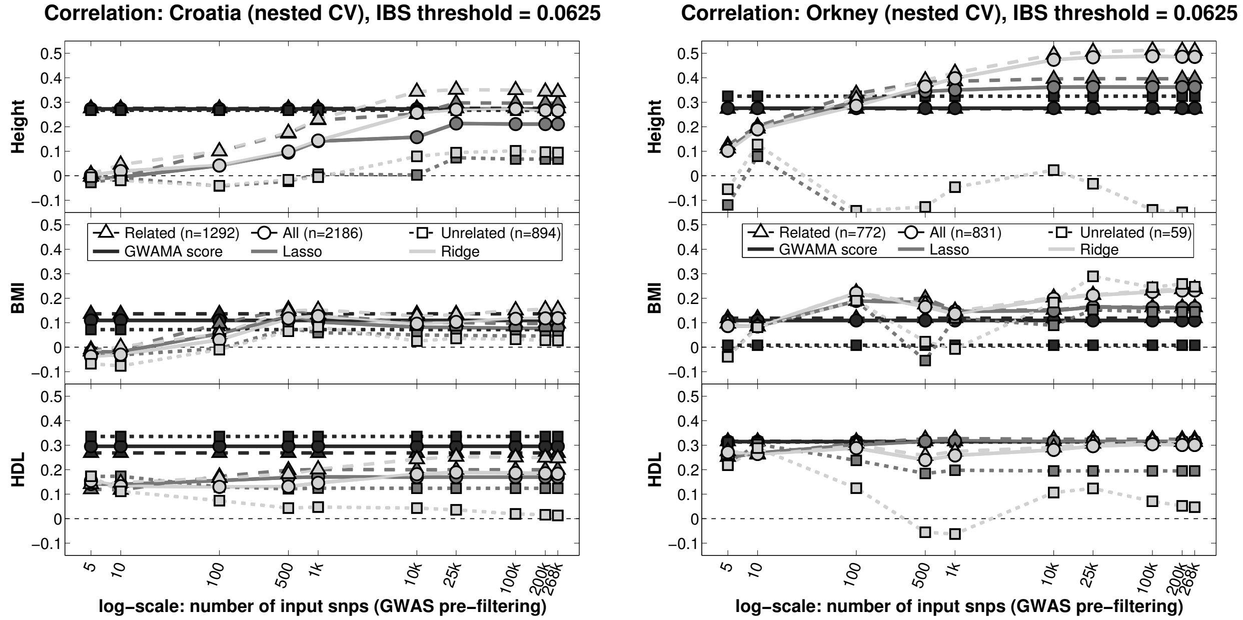


**Supplementary Figure S8.** Accuracy of LASSO and RR in within-cohort prediction computed using 'related', 'unrelated' and 'all' testing individuals (IBS threshold = 0.0625) together with accuracy of the GWAMA-based polygenic score for the same individuals. The 'related' group (triangles) contains testing individuals with at least one nominal relative at the training data (IBS $\geq$ 0.0625). The 'unrelated' group (squares) contains testing samples that are nominally unrelated to all the training samples. The 'all' group (circles) contains all the testing samples. y-axis: accuracy is measured using the Pearson's correlation coefficient between the predicted and true phenotype of the corresponding testing samples. x-axis: an increasing number of SNPs are given as input to the regression models (plotted in log-scale). The SNP selection is performed by GWAS pre-filtering using the training data in each case. (Left): nested cross-validation in Croatia. (Right): nested cross-validation in Orkney.
