# Supplementary material for: Genomic prediction of complex human traits: relatedness, trait architecture and predictive meta-models
Source: Hum Mol Genet. 2015 Apr 26;24(14):4167–82. doi: 10.1093/hmg/ddv145 (PMC4476450; doi:10.1093/hmg/ddv145)
Supplement: Supplementary Data [file supp_ddv145_ddv145supp_table1.pdf]

Supplementary Table 1. Accuracy of SNP-based pedigree in within-cohort prediction.
We construct the SNP-based pedigree by thresholding the whole-genome Identity-By-State matrix, and setting values below the threshold to 0.
We compute the whole-genome Identity-By-State between individuals i and k by $IBS_{ik} = \frac{1}{P}\sum_{j=1}^{P}\frac{(x_{ij}-2p_j)(x_{kj}-2p_j)}{2p_j(1-p_j)}$, where p_j is the minor allele frequency of SNP j, $x_{ij} \in \{0,1,2\}$ is the genotype of individual i at SNP j, and $P = 267,912$ $(260,562$ in Orkney) is the number of the genotyped SNPs.
Using each thresholded IBS similarity matrix we train and evaluate a kernel Ridge Regression model (equivalent to GBLUP).
We report predictive performance on the testing data from the within-cohort nested cross-validation data partition in Croatia and in Orkney.

Cohort	Measure	Trait	IBS*2	IBS > 1/32	IBS > 1/16	IBS > 1/8	Best Pen. Reg. *3
Croatia	Correlation	Height	0.2621	0.2443	0.2377	0.2291	0.2725
Croatia	Correlation	BMI	0.1060	0.1068	0.1131	0.1263	0.1271
Croatia	Correlation	HDL	0.1822	0.1834	0.1562	0.1534	0.1890
Croatia	MSE*1	Height	0.9684	0.9736	0.9764	0.9808	0.9559
Croatia	MSE	BMI	1.0820	1.0613	1.0512	1.0432	1.0033
Croatia	MSE	HDL	1.0195	1.0064	1.0246	1.0242	1.0053
Orkney	Correlation	Height	0.4731	0.4687	0.4264	0.1310	0.4838
Orkney	Correlation	BMI	0.2241	0.2208	0.2133	0.1763	0.2315
Orkney	Correlation	HDL	0.2790	0.2700	0.2362	0.1364	0.3087
Orkney	MSE	Height	0.7907	0.7957	0.8568	3.8539	0.7940
Orkney	MSE	BMI	1.0286	1.0393	1.0711	2.0088	1.0091
Orkney	MSE	HDL	0.9865	0.9999	1.0672	3.0274	0.9493

*1 *MSE*: Mean Squared Error.
*2 *IBS*: Prediction performance using the IBS similarity matrix without thresholding.
*3 *Best Pen. Reg.*: Prediction performance of the optimal penalised regression model on the same testing data.
The type of shrinkage and number of input SNPs for the optimal penalised regression model in each trait and cohort are given in Table 3.
